# FOXP2-immunoreactive corticothalamic neurons in neocortical layers 6a and 6b are tightly regulated by neuromodulatory systems

**DOI:** 10.1016/j.isci.2024.111646

**Published:** 2024-12-19

**Authors:** Guanxiao Qi, Danqing Yang, Fernando Messore, Arco Bast, Felipe Yáñez, Marcel Oberlaender, Dirk Feldmeyer

**Affiliations:** 1 Institute of Neuroscience and Medicine 10, Research Centre Jülich, 52425 Jülich, Germany; 2 Department of Psychiatry, Psychotherapy and Psychosomatics, RWTH University Hospital, 52074 Aachen, Germany; 3 In Silico Brain Sciences Group, Max Planck Institute for Neurobiology of Behaviour – Caesar, 53175 Bonn, Germany; 4 International Max Planck Research School (IMPRS) for Brain and Behavior, 53175 Bonn, Germany; 5 International Max Planck Research School (IMPRS) for Intelligent Systems, 72076 Tübingen, Germany; 6 Department of Integrative Neurophysiology, Center for Neurogenomics and Cognitive Research, Vrije Universiteit Amsterdam, 1081 Amsterdam, the Netherlands; 7 Jülich-Aachen-Research Alliance ‘Brain’ - Translational Brain Medicine, Aachen, Germany

**Keywords:** Molecular biology, Neuroscience

## Abstract

The *FOXP2*/*Foxp2* gene, linked to fine motor control in vertebrates, is a potential candidate gene thought to play a prominent role in human language production. It is expressed specifically in a subset of corticothalamic (CT) pyramidal cells (PCs) in layer 6 (L6) of the neocortex. These L6 FOXP2+ PCs project exclusively to the thalamus, with L6a PCs targeting first-order or both first- and higher-order thalamic nuclei, whereas L6b PCs connect only to higher-order nuclei. Synaptic connections established by both L6a and L6b FOXP2+ PCs have low release probabilities and respond strongly to acetylcholine (ACh), triggering action potential (AP) trains. Notably, L6b FOXP2− PCs are more sensitive to ACh than L6a, and L6b FOXP2+ PCs also react robustly to dopamine. Thus, FOXP2 labels L6a and L6b CT PCs, which are precisely regulated by neuromodulators, highlighting their roles as potent modulators of thalamic activity.

## Highlights

• All L6 FOXP2+ pyramids innervate thalamus; L6b FOXP2+ cells target only higher-order nuclei
• L6a and L6b FOXP2+ cells form facilitating synapses, enabling profound signal amplification
• Strong cholinergic response in L6a and L6b FOXP2+ CT cells via α4β2α5 nicotinic receptors
• Robust dopamine D1 response in L6b FOXP2+ cells, indicating a unique modulatory mechanism

## Graphical abstract

**Figure:**
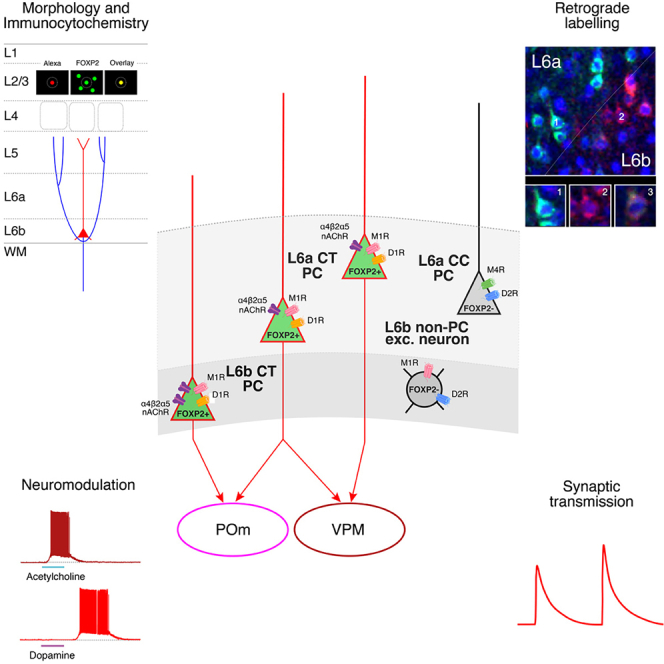

## Introduction

The forkhead box transcription factor P2 (*FOXP2*) gene (in humans *FOXP2*, in rodents *Foxp2*) is a highly conserved transcription factor present across many vertebrate species. It plays a crucial role in the neuronal circuits controlling vocalization and language, particularly in mammals and birds.^1^^,^^2^^,^^3^ Mutations in the *FOXP2* gene are associated with defective speech development and significant difficulties in controlling vocal-motor actions^1^^,^^2^^,^^4^ (for reviews see Enard; Vernes et al.; Graham and Fisher^5^^,^^6^^,^^7^). Additionally, in humans, FOXP2 has been implicated in various neuropsychiatric disorders such as autism spectrum disorder, attention-deficit hyperactivity disorder (ADHD), and schizophrenia.^8^^,^^9^^,^^10^^,^^11^^,^^12^^,^^13^^,^^14^ These disorders may result from dysfunctional neuronal networks caused by disruptions in the interaction between FOXP2 and its transcription targets.

FOXP2 regulates the transcription of numerous downstream target genes.^15^^,^^16^^,^^17^ It is involved in several neurodevelopmental processes, including neurite outgrowth, neuronal subtype specification, and the formation of synaptic circuits.^6^^,^^18^^,^^19^^,^^20^^,^^21^^,^^22^ Moreover, in rodents, *Foxp2* is critical for thalamocortical patterning and the proper formation of thalamocortical projections.^23^^,^^24^ Although *Foxp2* deletion in the neocortex does not lead to obvious histoarchitectural deficits, it does result in behavioral impairments, such as difficulties in motor skill learning, which may indicate impaired neuronal microcircuit formation.^25^^,^^26^^,^^27^ In humans, single nucleotide polymorphisms in *FOXP2* have been linked to ADHD, potentially due to synapse formation deficits.^11^

*Foxp2* is expressed in almost all neural networks involved in sensorimotor integration, modulation, and feedback control of fine motor output, including the olivocerebellar loop and corticobasal ganglia networks, and is also found in neurons of several thalamic nuclei and all neocortical areas.^22^^,^^28^^,^^29^^,^^30^ *Foxp2* expression is layer and neuronal cell-type specific and restricted to a subset of excitatory projection neurons that are almost exclusively located in layers 6a (L6a) and 6b of rodent neocortex.^31^^,^^32^^,^^33^ In layer 6a, *Foxp2* expression is indicative of corticothalamic (CT) projection neurons^12^^,^^27^^,^^34^^,^^35^ that co-express the neurotensin receptor 1 (*Ntsr1*) gene. Transcriptomic data suggest the presence of multiple L6 *Foxp2*-expressing glutamatergic neuron types.^36^ In certain neocortical areas, particularly in the premotor and medial motor cortices, *Foxp2* is also found in a subset of deep L5 pyramidal cells (PCs), albeit at low density, which show, however, pyramidal tract projections.^27^^,^^32^^,^^37^ Furthermore, in mouse medial prefrontal cortex (mPFC), at least a subset of *Foxp2*-expressing L6 PCs projects to the ventral tegmental area, which in turn sends dopaminergic afferents to the neocortex and is involved in numerous cognitive processes including decision-making and working memory.^38^

The expression of *Foxp2* is tightly regulated by neuromodulators such as acetylcholine (ACh) and dopamine. *Foxp2* expression coincides with the expression of the *Chrna5* gene, which encodes the α5 subunit of the nicotinic acetylcholine receptors (nAChRs) and selectively enhances the activity of L6 CT neurons.^39^^,^^40^^,^^41^ The nAChR α5 subunit conveys a higher ligand efficacy, increased Ca^2+^ permeability, and reduced receptor desensitization, suggesting that L6 CT neurons exhibit a pronounced nAChR response.^42^^,^^43^ It has also been proposed that type 1 dopamine receptors regulate the activity of L6 CT PCs.^44^ Furthermore, studies have demonstrated a reduced expression of the dopamine receptor 1 gene (*Drd1*) in neonate and adult *Foxp2* knockout mice in excitatory and inhibitory neurons in the frontal cortex.^12^ This suggests that FOXP2 may play a significant role in the development of dopamine-modulated cortical circuits. Hence, the CT microcircuitry in cortical L6 is regulated by at least two neuromodulatory systems involved in motor control^45^ and motor learning.^46^

The present study aimed to investigate the morphology, electrophysiology, synaptic properties, and neuromodulation of both FOXP2-immunopositive (FOXP2+) and FOXP2-immunonegative (FOXP2−) PCs in the rat primary somatosensory (S1) cortex. In layer 6, FOXP2+ PCs exhibited three distinct CT projection patterns: a projection solely to the first-order ventral posterior medial thalamic nucleus (VPM), a projection to both VPM and the higher-order posterior medial complex (POm), and a projection solely to the POm. Almost all POm-projecting neurons were located in deep layer 6, i.e., layer 6b. Corticocortical (CC) excitatory neurons in layer 6a and 6b were consistently FOXP2−. Furthermore, cholinergic and dopaminergic neuromodulation of L6a and L6b excitatory neurons correlates with the *Foxp2* expression pattern and the neuronal location within layer 6. Our findings indicate that FOXP2 is a marker for L6a and L6b CT PCs whose output is under tight control of cholinergic and dopaminergic neuromodulatory systems. These systems play crucial roles in motor behavior, arousal, and vigilance.

Here, the nomenclature given in the HUGO Gene Nomenclature Committee (HGNC; https://www.genenames.org/) and the Rat Genome Database (https://rgd.mcw.edu/) is used, i.e., the genes in humans and rodents are named *FOXP2* and *Foxp2*, respectively, and the gene product (i.e., the protein) is named FOXP2 in all species.

## Results

### Expression of FOXP2 in cortical and subcortical areas

**Figure 1:**
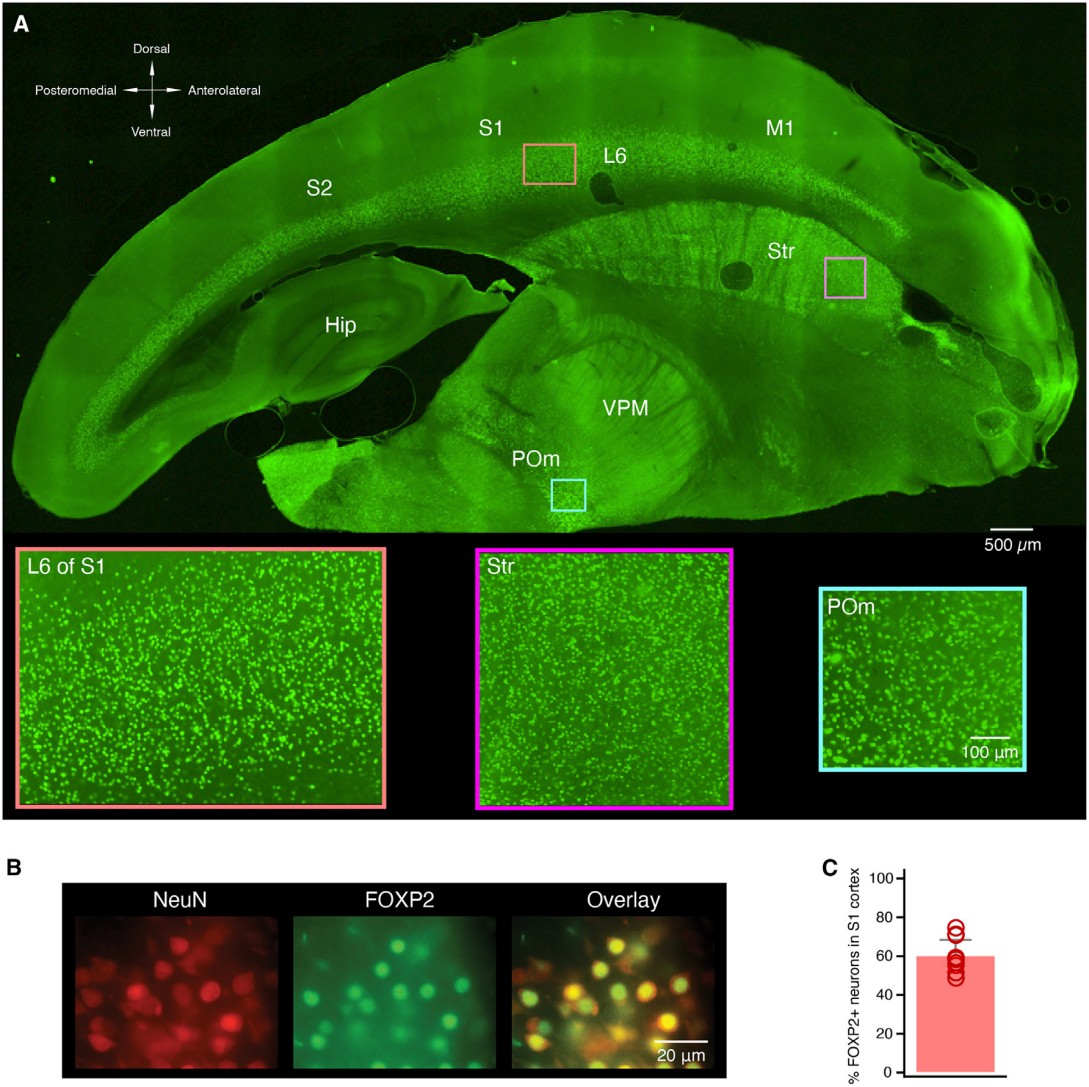
FOXP2 immunoreactivity in the neocortex (A) FOXP2 immunofluorescence in a 150 μm thalamocortical rat brain slice. Insets show the high density of FOXP2 immunoreactivity in cortical layer 6 (L6), the striatum (Str), and the posterior medial nucleus of the thalamus (POm). (B) Co-localization of NeuN and FOXP2 in cortical layer 6 at higher optical resolution. (Left) L6 neurons that were immunoreactive for NeuN (left) and FOXP2 (middle); the right panel shows the overlay of NeuN and FOXP2 labeling. (C) Relative proportion of FOXP2+ neurons in L6 in S1 barrel cortex. The bar graph shows the proportion of FOXP2+ neurons relative to the total number of neurons in layer 6 (*n* = 12 slices). Data are represented as mean + standard deviation. In layer 6, 60% of excitatory neurons are immunoreactive for FOXP2.

In the neocortex, the expression of the *Foxp2* gene is a highly specific marker of cortical layer 6.^31^^,^^32^ Here, FOXP2 immunoreactivity was analyzed across cortical and subcortical areas in 150 μm thick thalamocortical slices (Figure 1). FOXP2-related immunofluorescence was found exclusively and uniformly in layer 6 of the entire neocortex including the peri- and entorhinal cortex. In the primary somatosensory cortex (S1), 60.4 ± 8.0% of neurons (*n* = 12 slices) were FOXP2-positive (Figures 1B and 1C).^12^^,^^27^^,^^35^ In adjacent cortical areas, i.e., the secondary somatosensory cortex (S2) and primary motor cortex (M1), FOXP2 labeling was predominantly observed in layer 6 and to a markedly lesser extent in deep layer 5 (Figure 1A). In subcortical areas, the FOXP2 signal was robust in the striatum and in particular in the POm (98.8 ± 2.5% of FOXP2+ neurons (*n* = 9 slices; Figure S1), but only scattered in the VPM, and virtually absent in the hippocampus (Figure 1A).

### Cell-specific morphological properties of FOXP2+ and FOXP2− excitatory neurons in layer 6a and 6b

To investigate the morphological characteristics of FOXP2+ and FOXP2− neurons in S1 barrel cortex, we performed whole-cell patch-clamp recordings combined with *post hoc* FOXP2 immunolabeling and biocytin staining. In total, 171 L6 excitatory neurons and ten L6 interneurons were recorded, of which 62 excitatory neurons were located in upper layer 6 (layer 6a, defined here as the upper 70% of layer 6) and 97 in layer 6b (Figure S2); the remaining 12 excitatory neurons were excluded from the analysis because of an ill-defined location (either close to the L5-L6 border or to the L6a-L6b border). After FOXP2 immunolabeling, neurons with high-quality biocytin staining (*n* = 40) were carefully selected for detailed three-dimensional (3D) morphological reconstructions.

**Figure 2:**
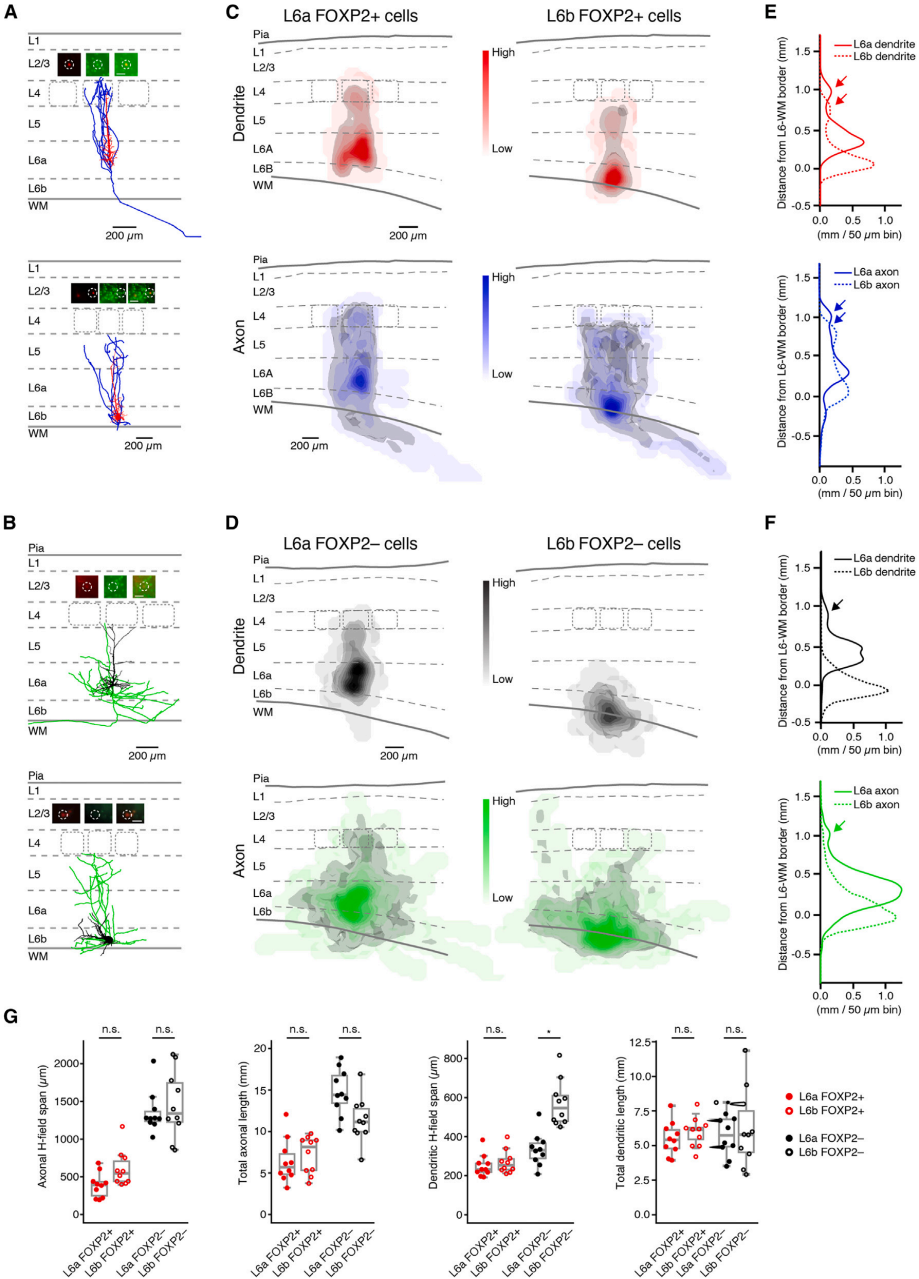
Morphological differences between L6a and L6b FOXP2+ and FOXP2− excitatory neurons (A) Representative axo-somatodendritic morphology of a L6a FOXP2+ PC (top) and L6b FOXP2+ PC (bottom). Dendrites are in red and axons in blue. The insets illustrate the FOXP2 immunoreactivity (see STAR Methods for details). In all insets, the left panel shows the fluorescent biocytin-conjugated Alexa 594 labeling of the recorded neuron, the middle panel displays the FOXP2 labeling, and the right panel presents an overlay of the biocytin and FOXP2 staining. The scale bar in the right panel represents 50 μm and applies to the other panels. (B) Representative axo-somatodendritic morphology of a L6b FOXP2− PC (top) and a L6b FOXP2− PC (bottom). Dendrites are in black and axons in green. Insets illustrate the FOXP2 immunoreactivity [for details see (A)]. (C) 2D projections of 3D density maps for L6a (left) and L6b (right) FOXP2+ PC dendrites and axons; color code as in (A). The 80 percentile of the integrated dendritic and axonal length density is shown in gray. (D) 2D projections of 3D density maps for L6a (left) and L6b (right) FOXP2− neuron dendrites and axons; color code as in (B). The 80 percentile of the integrated dendritic and axonal length density is shown in gray. (E) 1D dendritic (top, red) and axonal (bottom, blue) densities for L6a and L6b FOXP2+ PCs along the vertical axis. In these plots, 0 mm is the position of the L6/WM border. Solid lines represent L6a neurons and dashed lines L6b neurons. Arrows indicate the dendritic and axonal projections to superficial cortical layers. (F) 1D dendritic (top, black) and axonal (bottom, green) densities for L6a and L6b FOXP2− excitatory neurons along the vertical axis. In these plots, 0 mm is the position of the L6/WM border. Solid lines represent L6a FOXP2− neurons and dashed lines L6b FOXP2− neurons. Note that L6b FOXP2− excitatory neurons comprise a heterogeneous population of non-PC neurons with a dendritic and axonal domain that projects into the WM. Arrows indicate the dendritic and axonal projections to superficial cortical layers. (G) Boxplots showing the relative cortical depth, axonal horizontal field span, the total axonal length, and the horizontal dendritic field span for L6a FOXP2+ (*n* = 10), L6b FOXP2+ (*n* = 10), L6a FOXP2− (*n* = 10), and L6b FOXP2− (*n* = 10) excitatory neurons, respectively. The boxplot displays the interquartile range (IQR), with whiskers indicating 1.5 times the IQR and a horizontal line marking the median. The statistical significance was assessed using the Wilcoxon Mann-Whitney U test.

Figures 2A, 2B, S3, and S4 show the somatodendritic and axonal domains of representative FOXP2+ and FOXP2− excitatory neurons located in L6a and 6b, respectively. Without exception, FOXP2+ neurons in both layer 6a and 6b were found to be upright PCs with apical dendrites oriented toward to the pial surface. The dendritic domains of L6a and L6b FOXP2+ PCs were confined to their home barrel column and showed no significant differences (Figure 2; Tables 1 and S1). However, the apical dendrite of L6a FOXP2+ PCs predominantly terminated in layer 4, whereas that of L6b FOXP2+ PCs terminated in layer 5. Axon collaterals of L6a and L6b FOXP2+ PCs projected to either layer 4 or upper layer 5, respectively, depending on their location in layer 6 (Figures 2A, 2C, and 2E). Although axon collaterals of L6a FOXP2+ PCs were mainly confined to their home barrel column,^47^ those of L6b FOXP2+ PCs also innervated adjacent columns (horizontal field-span: 388.5 ± 163.6 μm for L6a FOXP2+ PCs [*n* = 10]; 607.1 ± 238.9 μm for L6b FOXP2+ PCs [*n* = 10]) (Figures 2A, 2C, 2E, and S3; Tables 1 and S1). The primary axons of L6a and L6b FOXP2+ PCs projected deeply into the white matter (WM) and turned laterally to run parallel to other axons.

**Table 1:** Morphological properties of L6 FOXP2+ and FOXP2− excitatory neurons

	L6a FOXP2+ (*n* = 10)	L6b FOXP2+ (*n* = 10)	L6a FOXP2− (*n* = 10)	L6b FOXP2− (*n* = 10)
**Soma**				

Depth (μm)	1479.0 ± 81.8	1700.6 ± 155.4	1384.4 ± 102.0	1739.6 ± 158.2
Relative depth (%)	77.7 ± 4.4	91.8 ± 2.5	72.7 ± 5.3	93.7 ± 2.5
perimeter (μm)	56.7 ± 7.7	54.8 ± 7.6	66.7 ± 11.4	63.5 ± 10.2
area (μm^2^)	242.6 ± 60.9	229.4 ± 61.3	340.8 ± 110.3	299.3 ± 95.0

**Dendrite**				

No. of apical dendrite branches	30.2 ± 7.2	26.9 ± 8.5	17.6 ± 9.2	N/A
Total length of apical dendrite (μm)	3634.2 ± 985.7	4009.4 ± 714.7	2485.5 ± 754.3	N/A
No. of basal dendrites	6.8 ± 1.4	7.2 ± 1.5	6.0 ± 1.6	6.0 ± 1.2
No. of basal dendrite branches	18.7 ± 3.1	20.4 ± 9.3	23.6 ± 8.8	36.3 ± 15.2
Total length of basal dendrites (μm)	1870.2 ± 391.8	2001.2 ± 520.9	3300.2 ± 1026.8	6222.4 ± 2804.9
Horizontal field span of dendrite (μm)	249.9 ± 57.0	269.6 ± 59.7	333.9 ± 84.0	570.6 ± 117.9
No. of dendritic branches	48.9 ± 8.5	47.3 ± 15.6	41.2 ± 15.6	36.3 ± 15.2
Total length of dendrites (μm)	5504.4 ± 1205.3	6010.7 ± 723.0	5785.7 ± 1530.2	6222.4 ± 2804.9

**Axon**				

Horizontal field span of axon (μm)	388.5 ± 163.6	607.1 ± 238.9	1353.5 ± 274.8	1453.6 ± 445.8
No. of axonal branches	26.3 ± 11.5	22.2 ± 8.2	49.1 ± 11.9	37.4 ± 9.5
Total length of axon (μm)	6404.7 ± 2637.6	7212.9 ± 2247.9	14769.9 ± 2779.7	11384.1 ± 2719.5

For statistical differences between FOXP2+ and FOXP2− neurons in layer 6a and 6b, see Table S1.

In marked contrast, the dendritic morphology of FOXP2− excitatory neurons was highly heterogeneous both within and between layers (Figures 2B, 2D, 2F, 2G, and S4; Table 1). The majority of L6a FOXP2− excitatory neurons were upright PCs with apical dendrites terminating either in layer 4 or 5. The dendritic arbor of L6a FOXP2− PCs was confined to the home column with a horizontal field-span of 333.9 ± 84.0 μm (*n* = 10). A bipolar or inverted dendritic morphology was observed in a minority of L6a FOXP2− excitatory neurons. Axon collaterals of L6a FOXP2− PCs projected horizontally in infragranular layers with an average field-span of 1353.5 ± 274.8 μm (*n* = 10). Furthermore, tall, putative claustrum-projecting PCs with a wide basal dendritic domain and an apical dendrite terminating in L1^48^^,^^49^^,^^50^ were also found to be FOXP2−.

In layer 6b, no upright FOXP2− PCs were found. L6b FOXP2− excitatory neurons comprised two main morphological subgroups: inverted pyramidal cells and multipolar spiny neurons.^51^^,^^52^ These neurons have long dendritic and axonal branches with a horizontal field-span of 570.6 ± 117.9 μm (*n* = 10) and 1453.6 ± 445.8 μm (*n* = 10), respectively (Figures 2B, 2D, 2F, 2G, and S4; Tables 1 and S1). L6b FOXP2− bipolar and horizontally/tangentially oriented pyramidal cells were only infrequently found. In addition to L6 excitatory neurons, 10 L6 interneurons with either fast spiking (*n* = 3) or non-fast spiking (*n* = 7) firing patterns were recorded. All were FOXP2− (Figure S5) and had diverse axonal domains, ranging from dense local to translaminar/transcolumnar projection patterns.

The average dendritic and axonal arborization pattern of FOXP2+ and FOXP2− excitatory neurons in layer 6 was determined by calculating 1D and 2D dendritic and axonal density maps (Figures 2C–2F). Basal dendrites of both L6a and L6b FOXP2+ PCs are mainly located in their respective layers. In addition, local dendritic density maxima were identified in the region where the apical dendrites terminated, i.e., at the L4/5A border and in the middle of L5 for L6a and L6b FOXP2+ CT PCs, respectively (Figures 2C and 2D; see also arrows in Figures 2E and 2F). The L6a FOXP2+ PC axon has only a narrow axonal domain and in S1 barrel cortex innervates only the home barrel column. In contrast, the axon field span of L6b FOXP2+ PCs is larger and innervates also neighboring barrel columns (Figures 2C and 2E).

L6a FOXP2+ and FOXP2− PCs have largely similar dendritic domains. In contrast, the dendritic domain of L6b FOXP2− excitatory neurons is markedly different from that of L6b FOXP2+ PCs, being almost exclusively located close to the L6b-WM border (Figures 2D and 2F). The axonal density profiles of both L6a and L6b FOXP2− excitatory neurons span several barrel columns in the horizontal direction, indicating a corticocortical axonal projection. Axons of L6a FOXP2− PCs innervate both granular and infragranular layers, whereas axons of L6b FOXP2− excitatory neurons project mainly within L6 and the WM (Figures 2D, 2F, and S4).

### Cell-specific electrophysiological properties of FOXP2+ and FOXP2− excitatory neurons in layer 6a and 6b

**Figure 3:**
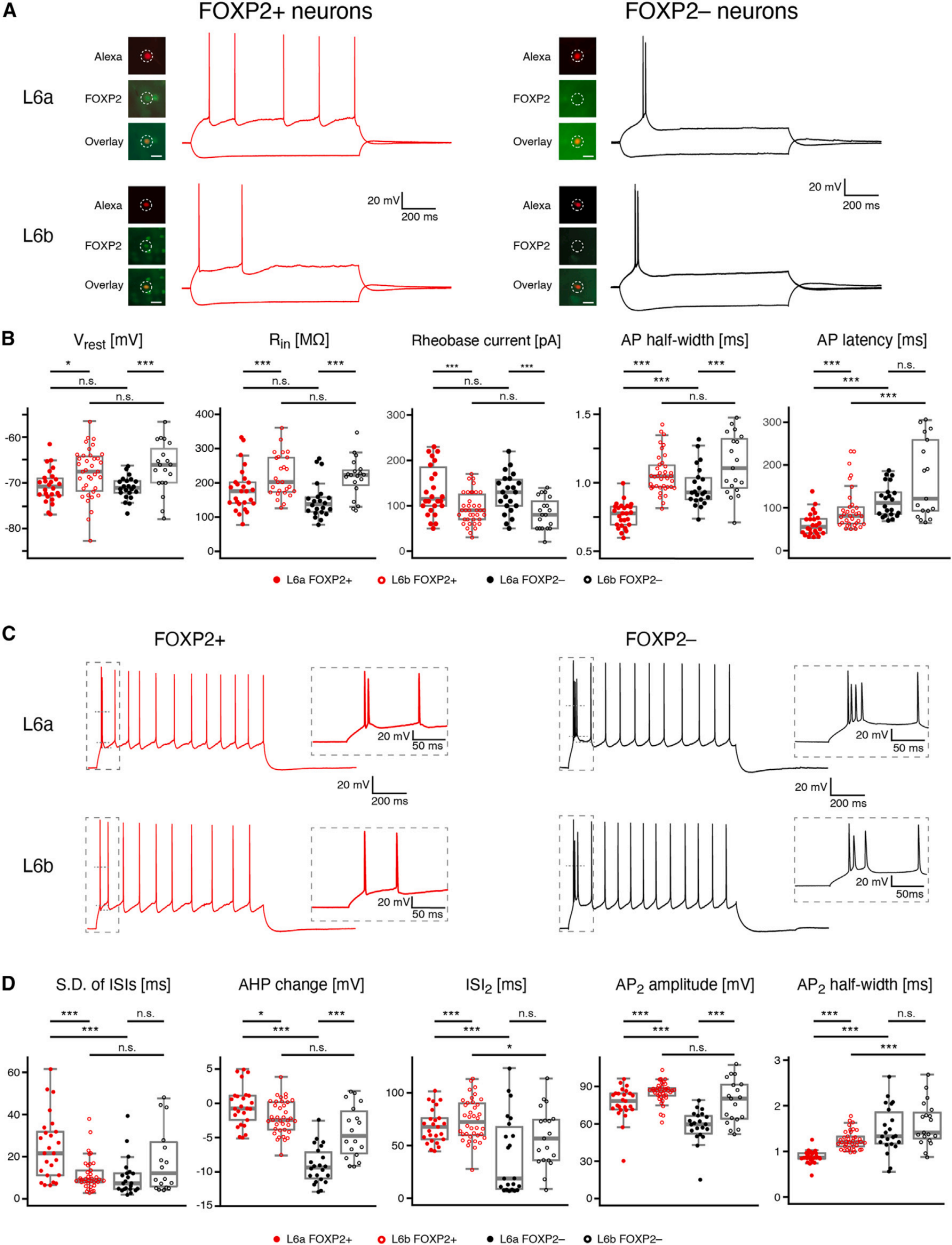
L6a and L6b FOXP2+ and FOXP2− excitatory neurons show distinct electrophysiological properties (A) Representative recordings of membrane potential changes in response to hyperpolarizing and rheobase current injections for FOXP2+ (left) and FOXP2− (right) excitatory neurons in layer 6a (top) and 6b (bottom), respectively. The insets illustrate the FOXP2 immunoreactivity (see STAR Methods for details). In all insets, the top panel shows the fluorescent biocytin-conjugated Alexa 594 labeling of the recorded neuron, the middle panel shows FOXP2 labeling, and the bottom panel presents an overlay of the biocytin and FOXP2 staining. The scale bar in the bottom panel represents 20 μm and applies to the other panels. L6a and L6b FOXP2+ PCs showed only one single AP near the rheobase, whereas FOXP2− excitatory neurons exhibited an initial burst of two to four APs. (B) Boxplots for passive electrophysiological properties and of the first AP of L6a FOXP2+ (*n* = 26, red closed circles), L6b FOXP2+ (*n* = 38, red open circles), L6a FOXP2− (*n* = 22; black closed circles), and L6b FOXP2– (*n* = 19, black open circles) excitatory neurons. (C) Representative recordings of repetitive firing patterns of L6a and L6b FOXP2+ (left) and FOXP2− (right) excitatory neurons. The insets in dashed boxes show an initial spike doublet (L6a and L6b CT PC) or AP burst (L6a and L6b CC neuron) at higher temporal resolution. (D) Boxplots for repetitive firing properties of L6a FOXP2+, L6b FOXP2+, L6a FOXP2−, and L6b FOXP2− excitatory neurons [labeling as in panel (B)]. In all plots, boxes indicate the interquartile range (IQR), the whiskers show the range of values that are within 1.5 times the IQR, and a horizontal line indicates the median. The statistical significance was assessed using the Wilcoxon Mann-Whitney U test.

The intrinsic membrane properties of L6a FOXP2+ (*n* = 26), L6b FOXP2+ (*n* = 38), L6a FOXP2–(*n* = 22), and L6b FOXP2− excitatory neurons (*n* = 19) including the passive electrophysiological properties and AP characteristics (Figure 3) were analyzed. L6a and L6b FOXP2+ excitatory neurons were found to be significantly different from L6a and L6b FOXP2− excitatory neurons in almost all electrophysiological properties (Figure 3; Tables 2 and S2). The rheobase current was significantly larger for L6a than for L6b FOXP2+ and FOXP2− PCs. At near-rheobase current injection, L6a and L6b FOXP2+ CT PCs generated a single AP that was sometimes followed by a short AP train. Under the same condition, L6a and L6b FOXP2− CC PCs produced a spike doublet or a short AP burst followed by a prominent depolarizing afterpotential (DAP; Figure 3A). The AP onset latency of L6a and L6b FOXP2+ excitatory neurons was shorter than in L6a and L6b FOXP2− excitatory neurons, respectively. The AP half-width and latency was shortest in L6a FOXP2+ excitatory neurons, whereas the AP threshold and AP amplitude did not differ significantly between the four excitatory neuron groups (Figure 3B; Table 2).

**Table 2:** Electrophysiological properties of L6 FOXP2+ and FOXP2− excitatory neurons

	L6a FOXP2+ (*n* = 26)	L6b FOXP2+ (*n* = 38)	L6a FOXP2− (*n* = 22)	L6b FOXP2− (*n* = 19)
**Passive**				

V_rest_ (mV)	−70.6 ± 3.6	−68.3 ± 6.3	−71.1 ± 2.5	−66.6 ± 5.6
R_in_ (MΩ)	180.7 ± 63.5	222.0 ± 57.0	149.5 ± 53.5	216.0 ± 58.2
τ_m_ (ms)	16.2 ± 4.4	20.6 ± 5.2	19.6 ± 4.2	23.7 ± 7.0
Sag (%)	12.9 ± 7.7	13.3 ± 7.1	10.6 ± 6.3	5.6 ± 4.4

**Single AP**				

Rheobase current (pA)	141.5 ± 76.4	93.7 ± 34.8	127.7 ± 43.7	83.4 ± 34.6
AP threshold (mV)	−36.4 ± 2.8	−37.5 ± 4.1	−37.5 ± 2.9	−37.6 ± 5.7
AP half-width (ms)	0.76 ± 0.09	1.07 ± 0.14	0.97 ± 0.15	1.13 ± 0.22
AP amplitude (mV)	91.9 ± 6.4	95.2 ± 5.7	90.3 ± 8.2	94.4 ± 10.2
AP latency (ms)	62.1 ± 27.0	97.6 ± 50.8	117.2 ± 37.9	166.9 ± 91.6
fAHP amplitude (mV)	10.1 ± 2.6	11.4 ± 4.5	3.4 ± 2.6	9.2 ± 5.7
fAHP latency (ms)	3.4 ± 2.6	27.2 ± 29.9	20.0 ± 36.2	16.5 ± 21.3

**Repetitive firing**				

Max. firing frequency (Hz)	24.8 ± 7.1	21.4 ± 5.6	23.5 ± 8.6	20.6 ± 4.4
Slope of F-I curve (APs/100 pA)	11.8 ± 4.1	15.4 ± 6.2	12.1 ± 4.5	15.5 ± 7.9
Adaptation ratio	1.1 ± 0.3	1.1 ± 0.2	1.1 ± 0.2	1.4 ± 0.5
SD of ISIs (ms)	24.6 ± 15.3	11.5 ± 7.6	10.2 ± 8.9	20.6 ± 20.1
ISI1 (ms)	19.2 ± 24.2	36.1 ± 20.7	6.7 ± 2.7	23.1 ± 15.4
ISI2 (ms)	68.6 ± 16.4	74.2 ± 19.9	40.6 ± 37.1	58.0 ± 28.1
ISI3 (ms)	87.7 ± 27.7	85.4 ± 14.1	83.5 ± 38.1	72.3 ± 22.0
AP1 amplitude (mV)	92.7 ± 5.7	94.8 ± 5.9	91.1 ± 5.7	92.8 ± 9.2
AP2 amplitude (mV)	76.7 ± 13.2	85.3 ± 8.3	56.4 ± 16.3	77.9 ± 17.4
AP3 amplitude (mV)	82.1 ± 9.2	86.2 ± 6.9	72.3 ± 14.5	84.3 ± 11.6
AP1 half-width (ms)	0.73 ± 0.08	1.00 ± 0.14	0.92 ± 0.11	1.05 ± 0.22
AP2 half-width (ms)	0.88 ± 0.14	1.25 ± 0.20	1.47 ± 0.52	1.60 ± 0.48
AP3 half-width (ms)	0.88 ± 0.12	1.24 ± 0.20	1.46 ± 0.38	1.51 ± 0.40
AP1 threshold (mV)	−40.1 ± 3.6	−39.2 ± 4.2	−38.6 ± 3.6	−38.2 ± 5.7
fAHP1 amplitude (mV)	6.3 ± 3.4	5.3 ± 3.5	0.0 ± 2.7	3.9 ± 4.6
AP9 threshold (mV)	−32.8 ± 4.4	−34.0 ± 4.9	−34.1 ± 4.4	−34.4 ± 6.7
fAHP9 amplitude (mV)	14.1 ± 2.0	12.5 ± 2.4	13.5 ± 1.7	11.8 ± 2.6
fAHP9−fAHP1 (mV)	−0.4 ± 2.9	−2.1 ± 2.4	−9.0 ± 2.7	−4.1 ± 3.8

For statistical differences between FOXP2+ and FOXP2− neurons in layer 6a and 6b, see Table S2.

Repetitive firing properties of FOXP2+ and FOXP2− excitatory neurons in layer 6a and 6b were analyzed using current pulses, which elicited ∼10 APs. In L6a and L6b FOXP2+ neurons, a single AP or a spike doublet occurred at the beginning of the AP train and was followed by irregular spiking, with variable inter-spike intervals (ISIs) (Figure 3C). In contrast, L6a and L6b FOXP2− excitatory neurons showed an initial burst of up to four APs, followed by regular spiking with constant ISIs. Furthermore, in FOXP2+ excitatory neurons, the AHP amplitude remained constant, whereas in FOXP2− excitatory neurons, AHPs became progressively larger during the spike train (Figure 3C). Furthermore, significant differences were observed in the duration of the 2^nd^ ISI, the amplitude, and half-width of 2^nd^ AP in a spike train between FOXP2+ and FOXP2− excitatory neurons in both layer 6a and 6b (Figure 3D; Table 2).

L6b FOXP2+ and FOXP2− excitatory neurons exhibited a largely similar dichotomy in firing patterns as L6a FOXP2+ and FOXP2− excitatory neurons, with the exception of a small subset of L6b FOXP2− excitatory neurons (see below). Specifically, L6b FOXP2+ excitatory neurons also displayed only an initial single AP or an AP doublet followed by an irregular AP firing with variable ISIs. In contrast, L6b FOXP2− excitatory neurons showed an initial burst of up to four APs with a subsequent regular spiking with constant ISIs (Figure S6). Of those, the majority of L6b FOXP2− excitatory neurons displayed a “rapidly adapting” firing pattern, with a long ISI between the initial burst and the following APs but shorter, stable ISIs thereafter. L6b FOXP2− excitatory neurons with a rapidly adapting firing patterns exhibit an inverted pyramidal morphology (Figures S6A and S6C). Another subpopulation of L6b FOXP2− excitatory neurons showed no initial AP burst but a slowly adapting firing pattern, with increasingly longer ISIs. These neurons had a multipolar dendritic morphology (Figures S6B and S6D). A thorough examination of the intrinsic membrane properties of the two populations of L6b FOXP2− excitatory neurons showed differences in input resistance (R_in_), 1^st^ AP latency, adaptation ratio, SD of ISIs, AHP change, and several other parameters (Figure S6E; Table S3).

In summary, L6a and L6b FOXP2+ PCs exhibit differences in several electrophysiological properties (e.g., R_in_, AP half-width, SD of ISIs; see Figure 3; Table 2) and their dendritic and axonal arborization pattern (Figure 2; Table 1). FOXP2− excitatory neurons in layer 6 comprise three morpho-electrophysiological phenotypes: L6a upright PCs with rapidly adapting (or burst spiking) firing patterns, L6a and L6b inverted PCs with rapidly adapting firing patterns, and L6b multipolar excitatory neurons comprising neurons with no apparent main dendrite or a tangential/oblique main dendrite^52^ and a slowly adapting firing pattern (Figures 2, 3, and S6; Tables 1 and 2).

### Synaptic connections established by L6a and L6b FOXP2+ and FOXP2− excitatory neurons have distinct functional properties

**Figure 4:**
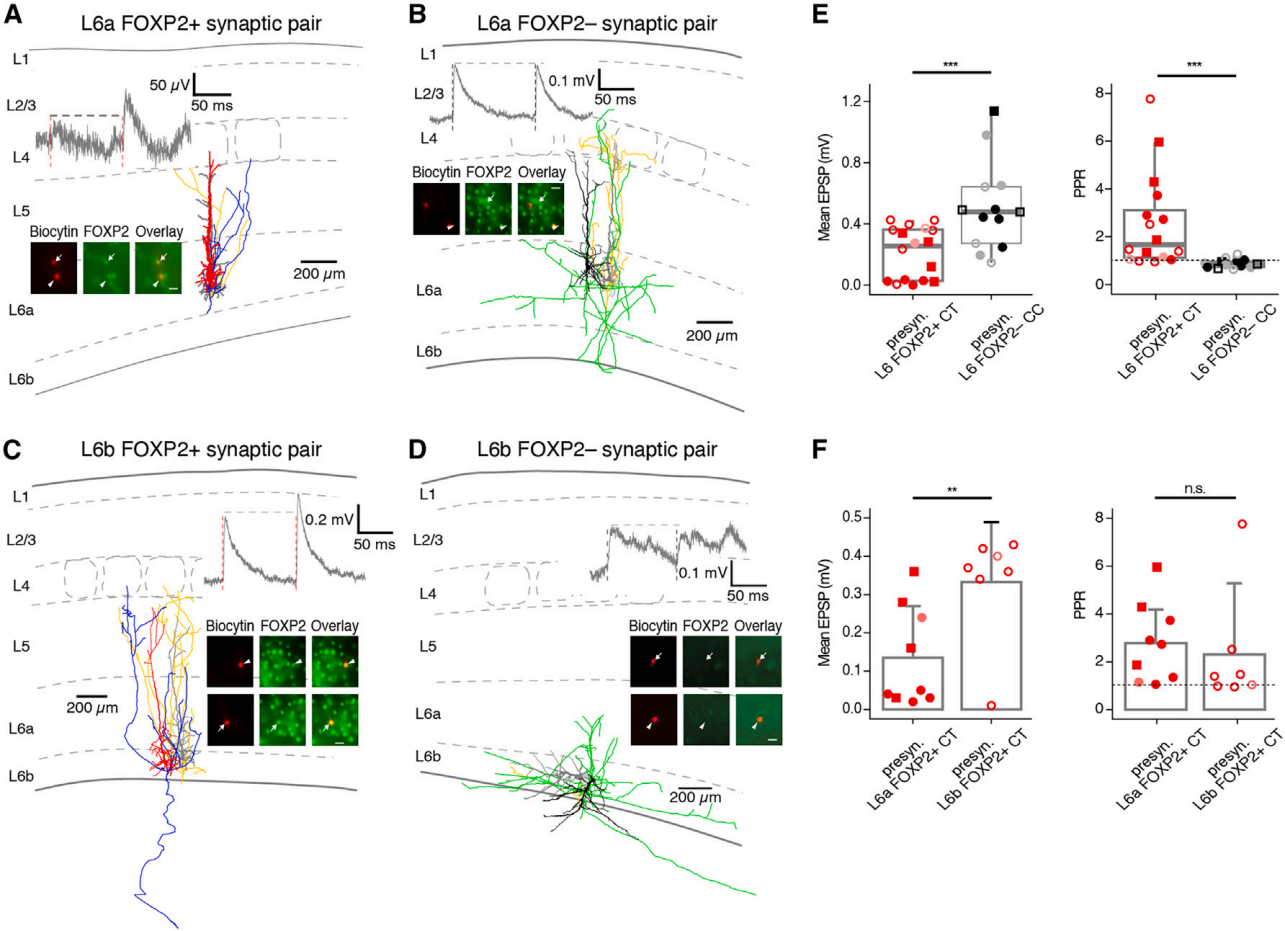
Synaptic connections established by L6 FOXP2+ and FOXP2− excitatory neurons show distinct properties (A–D) L6a and L6b synaptic connections; insets, FOXP2 immunoreactivity. Morphology, electrophysiology, and FOXP2 immunoreactivity for synaptic connections between (A) two L6a FOXP2+ CT PCs, (B) a L6a FOXP2− CC and a L6a FOXP2+ CT PC, (C) two L6b FOXP2+ CT PCs, and (D) two L6b FOXP2− inverted PCs. Color code: red dendrite/blue axon: L6a and L6b FOXP2+ CT PC, black dendrite/green axon: L6a and L6b FOXP2− CC excitatory neuron, gray dendrite/dark yellow axon: postsynaptic L6a and L6b excitatory neuron. The insets illustrate the FOXP2 immunoreactivity (see STAR Methods for details). In all insets, the left panel shows the fluorescent biocytin-conjugated Alexa 594 labeling of the recorded neuron, the middle panel displays the FOXP2 labeling, and the right panel presents an overlay of the biocytin and FOXP2 staining. The scale bar in the right panel represents 20 μm and applies to the other panels. (E) Boxplots for the 1st EPSP amplitude (top) and paired-pulse ratio (bottom) of synaptic connections established by L6 FOXP2+ CT (*n* = 16) and L6 FOXP2− CC (*n* = 13), respectively. Synaptic connections for which the presynaptic neuron was identified only on morphological grounds (axonal domain, cortical depth, and electrophysiological properties) have been included in the analysis. Filled and open symbols represent L6a and L6b neurons, respectively. For presynaptic L6 CT PCs, postsynaptic neurons are given as bright red circles for L6 (FOXP2+) CT PCs, light red circles for L6 (FOXP2−) CC neurons, and bright red squares for L6 interneurons. For presynaptic L6 CC neurons, postsynaptic neurons are colored black circles for L6 (FOXP2+) CT PCs, gray circles for L6 (FOXP2−) CC neurons, and black squares for L6 interneurons. Box plots display the interquartile range (IQR), with whiskers indicating 1.5 times the IQR abd a horizontal line marking the median. The dashed line in the right graph indicates a PPR = 1.0. *p* values were calculated using the Wilcoxon Mann-Whitney U test. (F) Bar charts for the 1st EPSP amplitude (top) and paired-pulse ratio (bottom) of synaptic connections established by L6a FOXP2+ CT (*n* = 9) and L6b FOXP2+ CT (*n* = 7), respectively. Synaptic connections for which the presynaptic neuron was identified only on morphological grounds (axonal domain, cortical depth, and electrophysiological properties) have been included in the analysis. Data are represented as mean + standard deviation. The dashed line in the right graph indicates PPR = 1.0. *p* values were calculated using the Wilcoxon Mann-Whitney U test.

Paired recordings from synaptically coupled L6 FOXP2+ and FOXP2− excitatory neurons were conducted to reveal potential differences in EPSP time course, neurotransmitter release probability, and synaptic dynamics. Following the electrophysiological recordings, FOXP2 antibody labeling and subsequent biocytin staining were performed Figures 4A–4D. A total of 29 synaptic connections were obtained. Nine were established by presynaptic L6a FOXP2+ PCs, seven by L6b FOXP2+ PCs, eight by L6a FOXP2− PCs, and five by L6b FOXP2− excitatory neurons. Postsynaptic neurons in these connections include both L6a and L6b FOXP2+ and FOXP2− excitatory neurons as well as L6b fast spiking (FS) and non-fast spiking (nFS) interneurons. All L6 connections with a presynaptic FOXP2+ PC were weak (1^st^ EPSP amplitude: 0.22 ± 0.17 mV, *n* = 16) and exhibited strong short-term facilitation (paired-pulse ratio EPSP_2_/EPSP_1_ [PPR]: 2.57 ± 1.99) Figures 4A, 4C, and 4E. This suggests a low initial synaptic release probability at these connections. Conversely, connections with a presynaptic FOXP2− neuron were comparatively strong (1^st^ EPSP amplitude for presynaptic L6 FOXP2– PCs: 0.51 ± 0.29 mV, *n* = 13) and showed short-term depression (PPR: 0.88 ± 0.19) Figures 4B, 4D, and 4E. Thus, synaptic connections established by presynaptic L6 FOXP2+ and FOXP2− excitatory neurons demonstrate highly distinctive synaptic efficacy and short-term plasticity, indicating a presynaptic cell-type specificity.

Furthermore, we observed a significant difference in the unitary EPSP amplitude of synaptic connections formed by L6a and L6b FOXP2+ CT PCs, with values of 0.13 ± 0.13 mV (*n* = 9) and 0.33 ± 0.15 mV (*n* = 7), respectively. However, no statistically significant difference was found for the PPR of L6a and L6b FOXP2+ CT PCs, with values of 2.78 ± 1.65 and 2.30 ± 2.47, respectively Figure 4F. This suggests that synaptic connections with presynaptic L6a FOXP2+ CT PCs and L6b FOXP2+ CT PCs have different synaptic properties, with the former showing a weaker synaptic strength compared to the latter, whereas their short-term synaptic plasticity remains similar.

### FOXP2-immunopositive neurons in layer 6a and L6b are corticothalamic neurons

**Figure 5:**
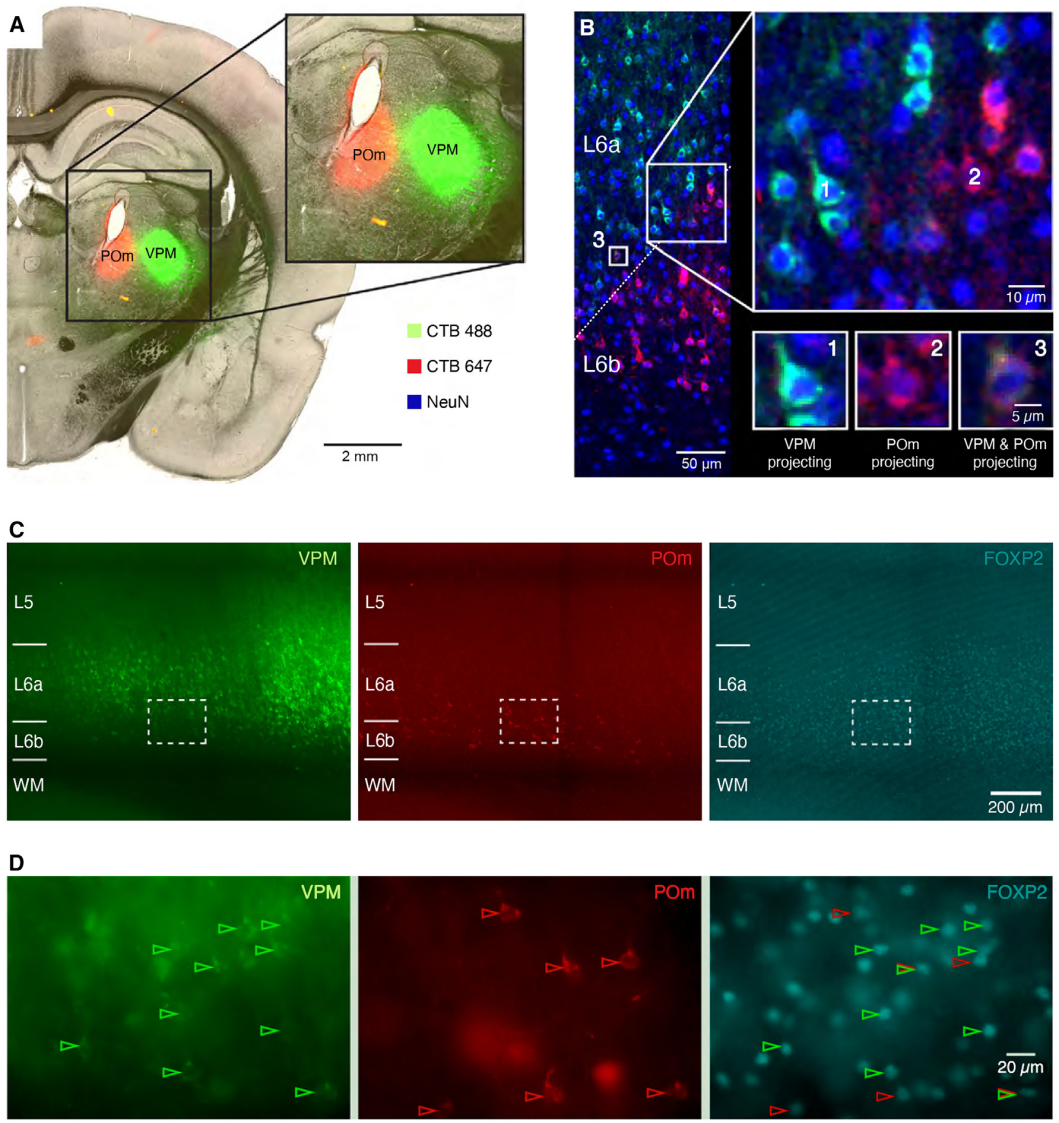
FOXP2+ neurons are corticothalamic (CT) neurons that project to the VPM or POm or both (A) Cholera toxin subunit B (CTb) conjugated with two Alexa Fluor dyes, A488 and A647, was separately microinjected into the VPM and POm thalamic nuclei. (B) Retrogradely labeled neurons project to either the VPM exclusively (“green label,” 1), the POm exclusively (“red label,” 2), or both the VPM and POm (3). NeuN fluorescence labeling is in blue. The oblique dashed line in the leftmost panel marks the border region between layer 6a and 6b. Note the projection target specificity of L6a and L6b CT neurons. (C) Neurons labeled retrogradely via the VPM or both the VPM and POm are located in the superficial and middle layers of layer 6, respectively, as indicated by the FOXP2 labeling (on the right). The majority of L6 PCs that project exclusively to POm is situated in deep layer 6/layer 6b. (D) The areas enclosed by rectangles in (C) are shown as enlarged images. Green arrows mark L6 neurons projecting to VPM and red arrows those projecting to POm; overlay of red and green arrows indicates neurons projecting to both thalamic nuclei.

Layer 6 is the source of a high proportion of corticothalamic afferents.^51^^,^^53^ We investigated whether corticothalamic projections originated from FOXP2+ or FOXP2− PCs. To achieve this, choleratoxin subunit B (CTB) conjugated with Alexa Fluor 488 and 647 was injected in the same animal into the VPM and POm, respectively (Figure 5A).

The majority of neurons in layer 6, identified by the neuron-specific NeuN stain (Figure 5B), were retrogradely labeled by only one of the injected CTBs. Retrogradely labeled neurons projecting to the VPM were predominantly located in layer 6a (Figure 5C), whereas retrogradely labeled neurons projecting to the POm were predominantly located in layer 6b (Figure 5C). In the transition zone between layer 6a and 6b, a subpopulation of double retrogradely labeled neurons was observed, indicating that they innervate both the VPM and POm (Figure 5B). These neurons intermingled with those sending axonal projections to only one thalamic nucleus. All retrogradely labeled neurons in layer 6 were found to be FOXP2+, irrespective of whether they projected to the VPM, POm, or both (Figures 5C and 5D). In contrast, POm-projecting, retrogradely labeled neurons in layer 5 exhibited no FOXP2 immunoreactivity (Figure S7). Thus, FOXP2+ neurons represent all subpopulations of CT neurons in layers 6a and 6b but not layer 5.

### Differential cholinergic modulation of L6a and L6b FOXP2+ and FOXP2− excitatory neurons

**Figure 6:**
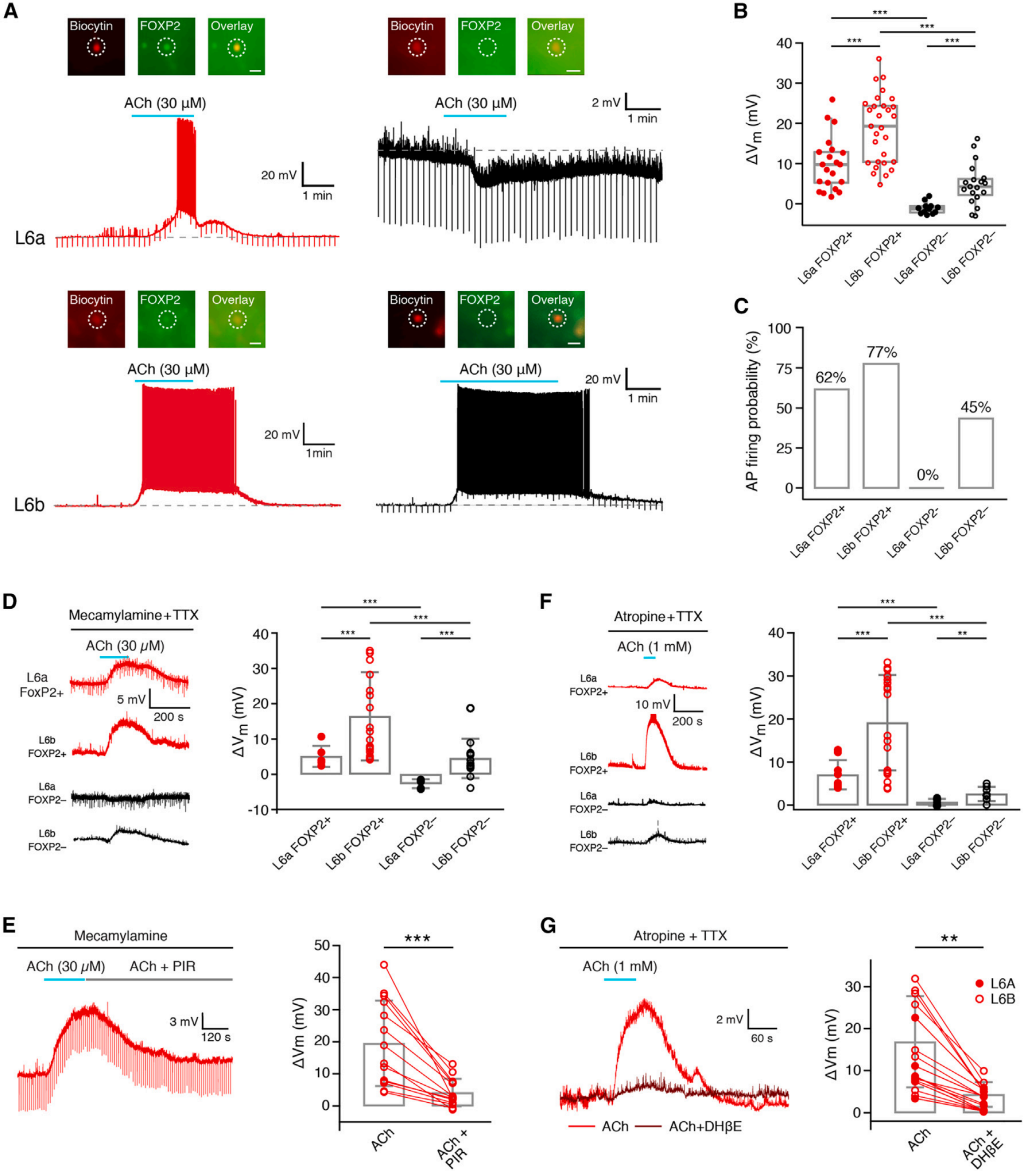
Cholinergic neuromodulation of L6a and L6b FOXP2+ and FOXP2− CT and CC excitatory neurons (A) Representative voltage responses of L6a (top row) and L6b (bottom row) FOXP2+ and FOXP2− excitatory neurons, respectively, to bathe-application of ACh (30 μM). The insets illustrate the FOXP2 immunoreactivity (see STAR Methods for details). In all insets, the left panel shows the fluorescent biocytin-conjugated Alexa 594 labeling of the recorded neuron, the middle panel displays the FOXP2 labeling, and the right panel presents an overlay of the biocytin and FOXP2 staining. The scale bar in the right panel represents 20 μm and applies to the other immunofluorescence images in this figure. (B) Boxplots for ACh-induced membrane potential changes in L6a FOXP+ CT (*n* = 21), L6a FOXP− CC PCs (*n* = 12), and L6b FOXP− CC excitatory neurons (*n* = 19) in the presence of 0.5 μM TTX to block AP firing. The ACh response of all four L6 excitatory neuron types was significantly different from one another. Boxes indicate the interquartile range (IQR), the whiskers show the range of values that are within 1.5 times the IQR, and a horizontal line indicates the median. (C) Fraction of different L6 excitatory neuron types firing action potential following application of 30 μM ACh in the absence of TTX. Note that L6a and L6b FOXP2+ CT PCs but also L6b FOXP2− CC excitatory neurons are highly responsive to ACh and show prolonged trains of spike trains. (D) The muscarinic component of the ACh response in L6 excitatory neurons was isolated by applying 30 μM ACh in the continuous presence of 1 μM mecamylamine and 0.5 μM TTX. L6a and L6b FOXP2+ CT PCs as well as L6b excitatory neurons showed an ACh-induced depolarization, whereas L6a FOXP2− CC excitatory neurons exhibited a hyperpolarizing response. Left, original recording of ACh-induced depolarizations in L6a and L6b FOXP2+ CT PCs and FOXP2− CC excitatory neurons; right, bar graphs showing the amplitude of the depolarization for the four different cell types. Data are represented as mean ± standard deviation. (E) Left, response of an L6 FOXP2+ CT PC to application of 30 μM ACh in the presence of 1 μM mecamylamine, a general nAChR antagonist. This ACh-induced depolarization was blocked by 0.5 μM pirenzepine, an M1 mAChR antagonist. Right, bar graphs show that pirenzepine blocked or largely reduced the mAChR-related depolarization induced by 30 μM ACh in recorded L6 FOXP2+ CT PCs. Data are represented as mean ± standard deviation. (F) The nicotinic component of the ACh response in L6 excitatory neurons was isolated using 200 nM atropine and 0.5 μM TTX. In L6 FOXP2+ CT PCs, 1 mM ACh elicited a large nAChR response, whereas in L6a and L6b FOXP2− CC excitatory neurons, no or only a small response, respectively, was observed. Left, original recordings of ACh-induced depolarizations in L6a and L6b FOXP2+ and FOXP2− (CT and CC) excitatory neurons; right, bar graphs showing the amplitude of the nAChR-induced depolarization for the four different cell types. Data are represented as mean ± standard deviation. (G) Left, response of an L6 FOXP2+ CT PC following application of 1 mM ACh in the presence of the general mAChR antagonist atropine (200 nM) and TTX (0.5 μM). The response was largely blocked by 10 μM DHβE, a specific antagonist of α4β2^∗^ subunit-containing nAChRs. Right, bar graphs show that DHβE blocked or largely reduced the nAChR-related depolarization induced by 1 mM ACh in recorded L6 FOXP2+ CT PCs. Data are represented as mean ± standard deviation. For statistical comparison of the data shown in (B), (D), and (E), the Wilcoxon Mann-Whitney U test was used and the data shown in (F) and (G), the Wilcoxon signed-rank test was used.

L6a and L6b CT PCs have been shown to express both nicotinic and muscarinic acetylcholine receptors (nAChRs and mAChRs).^54^^,^^55^ The effect of 30 μM ACh was tested in 81 L6 excitatory neurons located in both sublayers. In the presence of 0.5 μM TTX to block AP firing, ACh evoked a depolarization of 10.1 ± 6.6 mV (*n* = 21) in L6a FOXP2+ CT PCs and of 18.9 ± 8.4 mV (*n* = 29) in L6b FOXP2+ CT PCs (Figures 6A and 6B), with the ACh-induced depolarization being significantly larger in L6b than in L6a CT PCs (*p* < 0.001). In contrast, the ACh response showed greater variability in L6a and L6b FOXP2− neurons. In L6a CC FOXP2− excitatory neurons, 30 μM ACh resulted in either a hyperpolarization (10 out of 12; ΔV_m_: −1.4 ± 0.8 mV) or a weak depolarization (ΔV_m_: 1.2 and 2.1 mV, *n* = 2). The majority of L6b CC (FOXP2−) neurons showed an ACh-induced depolarization (6.3 ± 4.5 mV, *n* = 16 out of 19); in the remaining three, ACh caused a hyperpolarization (2.1 ± 1.0 mV; Figures 6A and 6B). In a separate series of experiments, the ACh response was investigated in the absence of TTX in the extracellular solution. In this instance, 30 μM ACh was observed to evoke AP firing in 62% of all L6a (FOXP2+) CT PCs, 77% of L6b (FOXP2+) CT PCs, and 45% of L6b (FOXP2−) CC excitatory neurons. However, no such response was evident in any L6a (FOXP2−) CC PCs (Figure 6C). Thus, L6 CT FOXP2+ PCs are highly responsive to ACh; in addition, L6b CC FOXP2− neurons show also a strong response to ACh.

To isolate the muscarinic component of the ACh response in L6 FOXP2+ and FOXP2− excitatory neurons, 30 μM ACh was applied in the presence of 0.5 μM TTX and 1 μM mecamylamine, a general nAChR antagonist. As illustrated in Figure 6D, L6a and L6b FOXP2+ CT PCs showed a depolarization of 5.1 ± 3.0 mV (*n* = 6) and 16.4 ± 12.5 mV (*n* = 20), respectively, following ACh application. Conversely, L6a FOXP2− CC excitatory neurons showed a hyperpolarizing response to ACh (−2.7 ± 1.3 mV, *n* = 5), consistent with previous findings,^55^ whereas L6b FOXP2− CC neurons exhibited a small depolarization (4.5 ± 5.6 mV, *n* = 12).

To determine the contribution of nAChRs to the ACh-induced response in L6a and L6b excitatory neurons, 1 mM ACh was bath-applied together with 200 nM atropine, a general mAChR antagonist, and 0.5 μM TTX. This resulted in a nAChR-mediated depolarization of 7.0 ± 3.4 mV (*n* = 9) and 19.1 ± 11.1 mV (*n* = 18) in L6a and L6b FOXP2+ CT PCs, respectively. On the other hand, the nAChR response in L6a and L6b FOXP2− CC neurons was comparatively small (0.7 ± 0.8 mV, *n* = 7 and 2.6 ± 1.6 mV, *n* = 8, respectively; see Figure 6F).

Antagonists for mAChR and nAChR subtypes were used to determine the specific receptor subtypes that contribute to the cholinergic response in L6a and L6b FOXP2+ PCs (Figures 6E and 6G). The muscarinic component was determined by applying 30 μM ACh, 1 μM mecamylamine, and 0.5 μM TTX. Subsequent co-application of ACh and 1 μM pirenzepine, an M1 mAChR antagonist, caused a reduction of the mAChR-mediated depolarization, indicating that this mAChR subtype mediates the muscarinic component of the ACh response (Figure 6E). As shown previously, the hyperpolarizing response observed in L6a CC neurons was mediated by M4 mAChRs.^55^

Because the nAChR-mediated response was negligibly small in L6 FOXP2− CC neurons, the nAChR subtype was only determined for L6 CT PCs. For this, ACh was applied in the presence of the specific antagonist for α4β2^∗^-subunit-containing nAChR DHβE (1 μM). In L6a and L6b FOXP2+ CT PCs, DHβE reduced the nAChR response from 10.0 ± 6.0 mV to 1.9 ± 2.4 mV and from 16.9 ± 10.9 mV to 4.3 ± 2.9 mV, respectively, suggesting that it was largely mediated by α4β2^∗^ nAChRs (Figure 6G). The data indicate that L6b FOXP2+ CT PCs are particularly responsive to ACh through activation of both M1 mAChRs and α4β2^∗^ nAChRs.

ACh caused an increase of neurotransmitter release at synapses established by L6a CT PCs but a decrease at L6a CC PCs 55; however, its effect at L6b excitatory synapses has not been characterized until now. We tested how 30 μM ACh affected synaptic transmission in four L6b excitatory neuron pairs (two pairs between L6b FOXP2+ CT PCs and two between L6b FOXP2− inverted PCs; Figure S8). At the two L6b CT connections, ACh caused a marked increase in the EPSP amplitude (14–99 μV and 0.42–0.58 mV) and a decrease in the paired-pulse ratio (6.3–0.4 and 1.0–0.5). In contrast, the EPSP amplitude at the two L6b inverted PC connections decreased in the presence of ACh (0.27–0.10 mV and 0.15–0.07 mV, respectively) and the paired-pulse ratio increased (1.1–2.2 and 0.6–2.2). Thus, as found for L6a PC connections, ACh selectively enhances the synaptic output of L6b CT PCs and reduced that of L6b CC neurons.

### Differential dopaminergic modulation of L6a and L6b FOXP2+ and FOXP2− excitatory neurons

**Figure 7:**
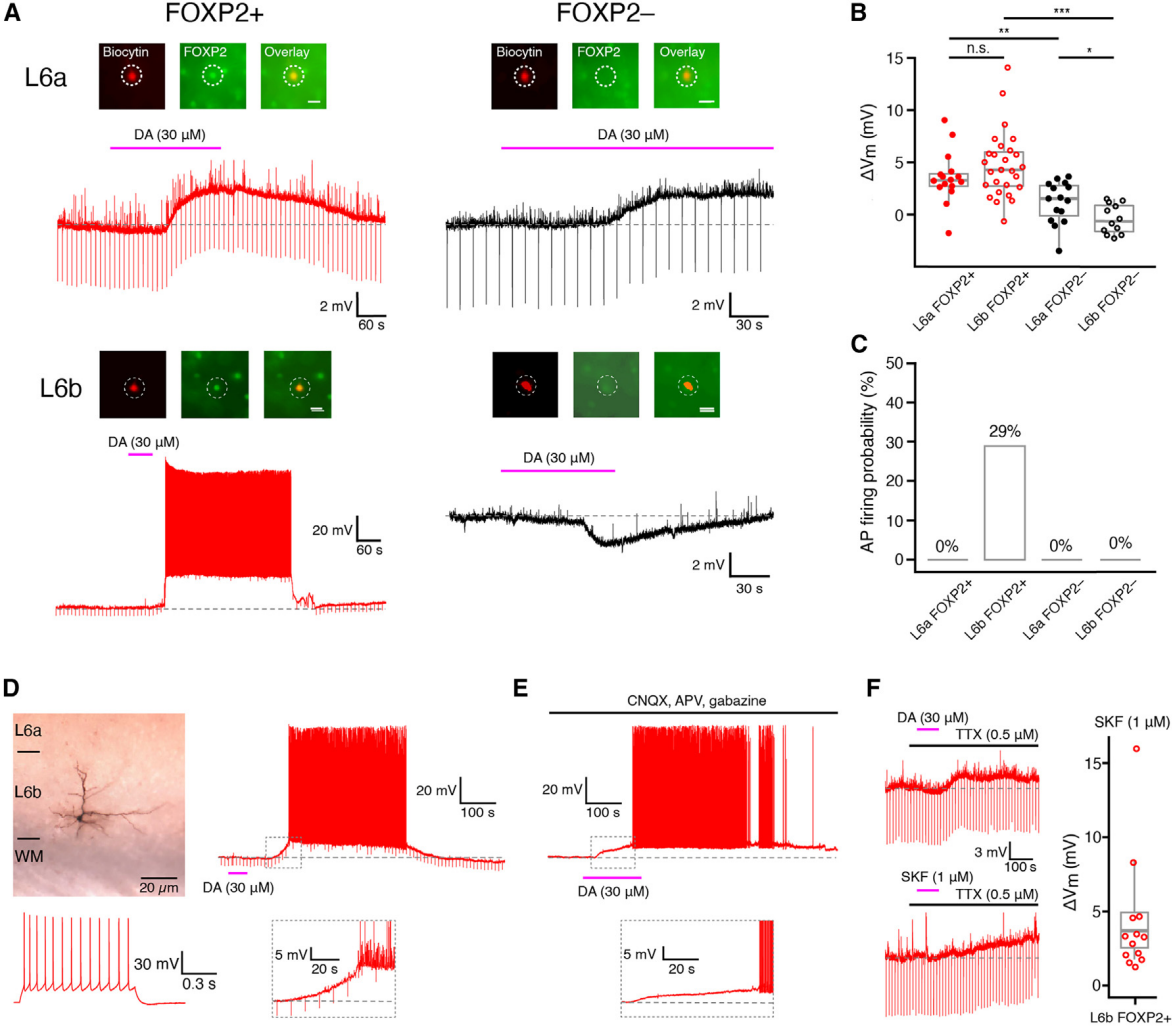
Neuromodulation of L6a and L6b FOXP2+ CT and FOXP2− CC excitatory neurons by dopamine (A) Representative voltage responses of L6a (top row) and L6b FOXP2+ and FOXP2− excitatory neurons (bottom row), respectively, to bathe-application of 30 μM dopamine (DA). The inset at the top shows the FOXP2 immunoreactivity. The insets illustrate the FOXP2 immunoreactivity (see STAR Methods for details). In all insets, the left panel shows the fluorescent biocytin-conjugated Alexa 594 labeling of the recorded neuron, the middle panel displays the FOXP2 labeling, and the right panel presents an overlay of the biocytin and FOXP2 staining. The scale bar in the right panel represents 20 μm and applies to the other panels. (B) Boxplots for DA-induced membrane potential changes in L6a FOXP2+ CT (*n* = 16), L6b FOXP2+ CT (*n* = 25), L6a FOXP2– CC (*n* = 15), and L6b CC (*n* = 12) excitatory neurons in the presence of 0.5 μM TTX to block AP firing. Boxes indicate the interquartile range (IQR), the whiskers show the range of values that are within 1.5 times the IQR, and a horizontal line indicates the median. For statistical comparison the Wilcoxon Mann-Whitney U test was used; ∗*p* ≤ 0.05, ∗∗*p* ≤ 0.01, ∗∗∗*p* ≤ 0.001; n.s., not significant. (C) Fraction of different L6 excitatory neuron types firing action potential following application of 30 μM DA in the absence of TTX. In 29% of L6b FOXP2+ CT PCs, 30 μM DA induced long-lasting spike trains; this was not observed in the other L6 excitatory neuron types. (D) L6b neurons that responded with prolonged AP firing following DA (30 μM) application were located close to the L6b-WM border and showed a regular firing pattern. (E) In a subset of L6b neurons (2 out of 9, i.e., 22%), DA-induced AP firing that was independent of glutamatergic and GABAergic synaptic activity. Note the delayed onset of AP generation. (F) Boxplots showing that the D1-like DA receptor agonist SKF (1 μM) elicited a depolarization (bottom left, see also the bar graph on the right) in all L6 excitatory neurons types with a DA-induced depolarization (top left, *n* = 13). The box indicates the interquartile range (IQR), the whiskers show the range of values that are within 1.5 times the IQR, and a horizontal line indicates the median.

The expression of the *Drd1* gene has been used as a marker for L6b CT PCs^56^^,^^57^^,^^58^^,^^59^^,^^60^ but so far dopaminergic responses from L6 CT and CC excitatory neurons have not been studied. Therefore, we examined whether L6a and L6b excitatory neurons are differentially modulated by dopamine (Figure 7).

Application of dopamine (30 μM) in the presence of TTX to L6 CT FOXP2+ excitatory neurons resulted in a depolarizing response, except for two L6 CT FOXP2+ PCs that showed a weak hyperpolarization (membrane potential change ΔV_m_: 3.4 ± 2.4 mV, *n* = 17 for L6a CT FOXP2+ PCs; 4.7 ± 3.2 mV, *n* = 28 for L6b CT FOXP2+ PCs). The effect of dopamine in FOXP2− excitatory neurons in layer 6 was heterogeneous (Figure 7B). In 11 out of 16 L6a CC FOXP2− excitatory neurons, dopamine caused a depolarization (ΔV_m_: 2.1 ± 1.2 mV) and in the remainder a weak hyperpolarization (ΔV_m_: −1.4 ± 1.2 mV, *n* = 5). Most L6b CC FOXP2− PCs (8 out of 13) dopamine induced a hyperpolarization (ΔV_m_: −1.7 ± 0.8 mV), whereas five showed a weak depolarizing response (ΔV_m_: 1.0 ± 0.5 mV).

When recorded in the absence of TTX, 30 μM dopamine induced a depolarization followed by prolonged high-frequency AP firing in 29% of L6b FOXP2+ PCs (see Figures 7A and 7C). These neurons exhibited a dendritic and axonal projection pattern similar to CT PCs, with apical dendrites mostly terminating in layer 6 (Figure 7D).^52^ They were located near the WM, which is significantly deeper than L6b neurons with subthreshold DA responses (relative depth in layer 6: 0.97 ± 0.20 and 0.92 ± 0.50, respectively) (Figure 7D). In all other L6 excitatory neurons, dopamine did not induce AP firing (Figures 7A and 7C). To investigate whether AP firing resulted from a dopamine-induced enhancement of synaptic input, dopamine was applied while blocking excitatory and inhibitory synaptic transmission with 10 μM CNQX, 50 μM AP5, and 10 μM gabazine. Under this condition, dopamine caused only a weak depolarization in the majority of L6b CT PCs. However, in two out of nine L6b CT PCs (i.e., 22%), AP firing was still observed in the presence of synaptic blocker (Figure 7E). This finding suggests that in these neurons the strong dopamine-induced depolarization is due to an intrinsic, postsynaptic mechanism independent of synaptic input.

To determine the receptor subtype mediating the dopamine-induced depolarization, we applied the D1-like dopamine receptor agonist SKF81297. L6a and L6b excitatory neurons, which showed a dopamine-induced depolarization, were also depolarized by a subsequent application of 1 μM SKF81297 (Figure 7F). In contrast, when dopamine application resulted in a hyperpolarization of an L6 excitatory neuron, SKF81297 showed no effect. When dopamine was applied together with 10 μM SCH23390, a D1 receptor antagonist, a dopamine-induced depolarization was no longer observed, suggesting that in layer 6, all FOXP2+ PCs and a subset of L6a and L6b FOXP2− CC neurons express D1-like receptors.

## Discussion

Here, we demonstrate distinct structural and functional properties of FOXP2+ and FOXP2− excitatory neurons in layer 6a and 6b of the primary somatosensory cortex. FOXP2 immunolabeling was found only in L6a and L6b CT PCs, whereas all other excitatory neurons in layer 6a and 6b were FOXP2−, including L6a CC PCs, tall, putative claustrum-innervating L6a PCs, or the diverse excitatory neuron types in layer 6b.^51^^,^^52^^,^^61^^,^^62^ FOXP2+ CT PCs and FOXP2− excitatory neurons were found to have different synaptic dynamics and were differentially modulated by ACh and dopamine. Notably, both neurotransmitters caused prolonged AP firing in L6b FOXP2+ CT PCs, underscoring their critical role in modulating corticothalamic feedback.

### CT PC types in neocortical layer 6

Our data suggest the existence of at least three distinct subtypes of CT PCs in layer 6 of the somatosensory barrel cortex. These subtypes all exhibit FOXP2 immunoreactivity but display differential innervation of the somatosensory thalamic nuclei. The first-order VPM nucleus relays sensory signals to the neocortex via the direct, “lemniscal” pathway and is only innervated by CT PCs located mostly in superficial layer 6a.^51^^,^^63^^,^^64^ The higher-order POm complex is part of the indirect “paralemniscal” pathway and receives inputs from three distinct PC types, namely deep L5 thick-tufted PCs,^65^^,^^66^ (for a review see,^67^) a subset of PCs in deep layer 6a that innervates both VPM and POm,^53^^,^^64^ and L6b PCs that exclusively project to POm.^53^^,^^57^^,^^58^

Deep L5 CT PCs form giant synapses with POm neurons where a single presynaptic AP can trigger a burst of postsynaptic APs, effectively “driving” POm neurons.^66^^,^^68^^,^^69^ These neurons also exhibit marked short-term synaptic depression. Extracortical synaptic connections between the brainstem and thalamus show similar synaptic dynamics and are also considered as thalamic “drivers.”^70^^,^^71^

In contrast, CT PCs in cortical layer 6 form small, “en passant” synaptic contacts with VPM and/or POm neurons. These CT synapses exhibit short-term synaptic facilitation and are of low efficacy.^53^^,^^63^^,^^68^ Therefore, CT PCs in layer 6 are considered to be “use-dependent” modulators of thalamic activity. It has been suggested that the underlying mechanism is the differential synaptic release probability at different CT synapses. This probability is high for “drivers” (i.e., deep L5 input to POm) but low for L6a and L6b inputs.^72^^,^^73^^,^^74^ Previous studies have demonstrated that in layer 6, presynaptic CT PCs determine the short-term plasticity and release probability of all their synapses, whether they are intracortical or corticothalamic.^75^^,^^76^ Therefore, it has been argued that synaptic dynamics of PCs generally reflect their long-range axonal projection targets.^77^^,^^78^ Consistent with this, we have shown that intracortical synaptic connections of L6a CT PCs, but not of CC PCs or corticoclaustral PCs, exhibit pronounced short-term facilitation.^50^ L6b CT PC synaptic connections with other neurons exhibit low synaptic efficacy, short-term synaptic facilitation, and a low release probability.^60^^,^^79^^,^^80^^,^^81^ This supports the notion that, like L6a CT PCs, L6b CT PCs modulate the activity of thalamic neurons.

### FOXP2 is a highly specific marker of CT neurons in layer 6a and 6b

*Foxp2* is expressed in layers 6a and 6b across all cortical areas. L6a FOXP2+ neurons in rodent primary somatosensory (S1), visual (V1), and auditory (A1) cortices are CT PCs, as shown by retrograde tracer injections into first-order thalamic nuclei.^27^^,^^34^^,^^82^^,^^83^ Single-cell reconstructions in rat S1 barrel cortex revealed that upper L6a PCs project exclusively to the VPM, whereas deep L6a PCs innervate both VPM and POm, the first- and higher-order thalamic nucleus.^51^^,^^53^^,^^63^^,^^64^^,^^84^

Our findings show that FOXP2 immunoreactivity is a selective marker for all three L6 CT PC types, regardless of their projection targets (VPM alone, both VPM and POm, or POm alone). In both layers 6a and 6b, FOXP2 specifically labels typical PCs with an apical dendrite and a narrow axonal domain but not “inverted” PCs or multipolar excitatory neurons with long-range axonal projections throughout layer 6 and deep layer 5^51^^,^^52^^,^^62^ or corticoclaustral PCs.^48^^,^^50^^,^^61^

The neurotensin receptor 1 gene, *Ntsr1*, is highly expressed in L6a CT PCs that project to either VPM alone or both VPM and POm.^64^^,^^85^ These two L6a CT PC types have been identified in earlier retrograde tracing studies.^51^^,^^63^ Transgenic *Ntsr1*-Cre mouse lines are utilized to identify L6a CT PCs in studies on cortico-thalamo-cortical (CTC) signaling.^86^^,^^87^^,^^88^^,^^89^^,^^90^^,^^91^^,^^92^ However, *Ntsr1* is also expressed in WM neurons located directly below layer 6b^93^ and in nucleus-accumbens-projecting L2/3 and L5a PCs.^38^ NTSR1-mediated depolarizations have been recorded in L6b excitatory neurons and fast-spiking interneurons,^94^^,^^95^ indicating that *Ntsr1* expression is not specific to L6a CT PCs.^95^^,^^96^ Our data show that FOXP2 does not label CC excitatory neurons, consistent with a previous study.^27^

In non-sensory cortical areas, *Syt6* (synaptotagmin 6 gene) is a more reliable CT neuron marker than *Ntsr1*.^97^ *Syt6* and *Ntsr1* show minimal overlap, suggesting they label specific subsets of L6a CT PCs. *Foxp2*, however, is expressed in layer 6 of all cortical areas, indicating its ubiquity as a marker for L6 CT neurons.

In layer 6b, a distinct population of CT PCs expressing *Drd1* (dopamine receptor 1 gene) projects exclusively to the higher-order POm nucleus^57^ and is highly sensitive to the wake-promoting neuropeptide orexin.^60^^,^^98^ We identified L6b FOXP2+ PCs that innervate exclusively POm and show a robust dopamine D1 receptor response, indicating co-expression of *Foxp2* and *Drd1*. Nonetheless, D1-receptor-mediated depolarizations are also observed in L6a CT PCs and a number of L6 CC excitatory neurons, suggesting that the expression of *Drd1* is not exclusive to L6b CT PCs.

### Cholinergic neuromodulation of FOXP2+ and FOXP2− excitatory neurons in layer 6a and 6b

Our findings highlight ACh as a potent modulator of both L6a and L6b FOXP2+ CT PCs, acting through muscarinic and nicotinic receptors to induce strong depolarizations even at micromolar concentrations. This modulation is critical during wakefulness, attention, and REM sleep, as ACh released from the basal forebrain and brainstem cholinergic nuclei can induce prolonged AP firing in almost two-thirds of L6a CT PCs and more than 75% of L6b CT PCs. In contrast, ACh consistently causes hyperpolarization in L6a FOXP2− CC PCs, whereas L6b FOXP2− CC excitatory neurons show moderately strong ACh-induced depolarizations. This differential response suggests a cell-specific cholinergic regulation of L6 neuron activity.

In addition, a recent study identified excitatory subplate neurons termed “ACh super-responders” due to their pronounced ACh-induced depolarizations,^99^ a feature resembling the ACh response of L6b CT PCs described here. Layer 6b is considered to be a remnant of the subplate,^100^^,^^101^^,^^102^^,^^103^ which suggests that L6b excitatory neurons retain the high ACh responsiveness akin to the “ACh super-responder” subplate neurons during early postnatal development.

*In situ* hybridization and antibody labeling studies have demonstrated that in layer 6, CT PCs predominantly express (α4)_2_(β2)_2_α5 nAChRs,^104^ a finding supported by several functional studies.^39^^,^^105^^,^^106^^,^^107^^,^^108^ This α4β2α5 nAChR is characterized by a high ACh sensitivity and a slow desensitization, resulting in a large and sustained ACh response in these neurons.^42^^,^^109^ In addition, α4β2α5 nAChRs exhibit a high Ca^2+^ permeability, which facilitates modulation of presynaptic neurotransmitter release and significantly increases synaptic efficacy. As previously demonstrated, ACh enhances synaptic release at L6a CT PC synapses, with the activation of presynaptic nAChRs being identified as a key mechanism in this process.^55^ The findings presented here indicate a comparable effect at the L6b CT pyramidal cell synapse.

Furthermore, the activation of postsynaptic nAChRs also significantly enhances the probability of AP firing in L6a and L6b CT PCs. The cholinergic modulation of L6 CT neurons demonstrated here implies that even relatively low ACh concentrations can efficiently recruit VPM and POm neurons, effectively modulating the gain of thalamocortical output. The prolonged firing can enhance thalamocortical feedback, crucial for sensory signal amplification during states of heightened attention.^110^ In particular, the strong ACh response in L6b FOXP2+ CT PCs suggests that they play a role in modulating thalamic activity dynamically, which could be essential for maintaining sensory signal fidelity during behavioral states requiring high attentiveness.

### Dopaminergic neuromodulation of FOXP2+ and FOXP2− excitatory neurons in layer 6a and 6b

Dopaminergic afferents to the neocortex originate mainly from the ventral tegmental area (VTA) and, to a lesser extent, the substantia nigra pars compacta.^111^^,^^112^ The somatosensory cortex receives dopaminergic inputs from mesocortically and mesocorticolimbically projecting VTA neurons.^113^ In rats, L6 CT PCs express dopamine D1 receptors as indicated by the presence of the dopamine- and cAMP-regulated neuronal phosphoprotein (DARPP-32).^114^ Dopamine D1-receptor-expressing neurons are primarily located in deep cortical layers 5 and 6,^44^^,^^115^^,^^116^^,^^117^ where the density of dopaminergic afferents is highest in rodents.^118^ However, in PFC, the majority of *Drd1*-expressing neurons were L6 CC neurons, including inverted PCs.^117^ In contrast, in the somatosensory cortex, the largest dopamine D1 receptor response was observed in L6b CT PCs, resulting in AP firing in nearly 30% of the tested neurons. A subset of L6 FOXP2− CC excitatory neurons also exhibited a weak depolarizing response to dopamine, which was smaller than in L6 FOXP2+ CT PCs. This indicates that *Drd1* expression is probably not exclusive to L6b CT PCs projecting to the POm. Nevertheless, the high expression of *Drd1* and the density of functional dopamine D1 receptors in L6b CT PCs may be the reason that this gene is considered to be a specific marker for these neurons.

The robust dopamine responses observed in L6b FOXP2+ CT PCs are consistent with the known roles of dopamine in modulating cortical activity related to cognition and attention.^45^^,^^119^^,^^120^ Our findings show that dopamine induces prolonged AP firing in L6b FOXP2+ CT PCs. This indicates that these neurons could play a significant role in attention regulation and sensory information processing, particularly through the POm. The capacity of dopamine to enhance the excitability of these neurons may facilitate a state-dependent modulation of sensory processing, thereby reinforcing the link between dopaminergic signaling and cognitive functions such as attention and working memory. However, the practical implications of dopaminergic input for CTC signaling are not yet fully understood and require further study.

### Role of L6a and L6b CT PCs in corticothalamic signaling and its neuromodulation

In the somatosensory system, L6a CT PCs innervate not only excitatory neurons in the VPM (upper layer 6a) or VPM and POm (lower layer 6a) but also inhibitory neurons in the thalamic reticular nucleus (TRN), which projects directly to both VPM and POm.^121^^,^^122^ As a result, L6a CT PCs modulate the activity of these thalamic nuclei by inducing direct, monosynaptic excitation and subsequent disynaptic inhibition through TRN neurons.^123^^,^^124^

In most studies on the effect of L6 CTC signaling, the focus is on L6a CT PCs. These L6a CT neurons exhibit diverse synaptic properties, integrating various inputs with distinct temporal dynamics, which allows them to modulate sensory processing based on behavioral states.^50^^,^^75^ L6 CT PCs affect both excitatory and inhibitory pathways, which can dynamically alter sensory processing based on context and behavioral states.^66^^,^^74^^,^^91^^,^^125^^,^^126^ In sensory cortices, CT feedback can alter the receptive fields of thalamic neurons, allowing for flexible adaptation to changing sensory environments and tasks.^127^^,^^128^^,^^129^ CT PCs in layer 6 also influence sensory processing by synchronizing thalamic and cortical activities,^130^^,^^131^^,^^132^^,^^133^ which is essential for integrating sensory information with cognitive and motor signals, and the initiation of complex behaviors and decision-making. Furthermore, CT feedback supports context-dependent processing by dynamically modulating thalamic activity based on cortical inputs and behavioral states. Therefore, neuromodulatory input to L6 CT neurons during different behaviors can significantly affect CT feedback by altering synaptic efficacy and response patterns^134^ that are crucial for gating cortical activity to the thalamus, allowing for context-dependent sensory processing.^135^

ACh and dopamine trigger the activation of L6a CT PCs, thereby enhancing VPM activity. However, ACh suppresses TRN activity through mAChRs,^123^ reducing net inhibition by L6 CT circuits.^136^ Additionally, ACh-induced L6b CT neuron activation recruits large L6 translaminar, fast-spiking interneurons whose axons span almost the entire cortical depth and primarily impact L2/3 and L5 PCs.^60^^,^^87^ Because of this complex neuronal circuitry, predicting the overall effect of ACh-induced firing in L6a CT PCs is challenging and requires further study because ACh enhances the excitability of several neuron types in layer 6.^137^^,^^138^

Stimulation of the posterior parietal cortex (PPC) enhances and prolongs cortical sensory signals, likely through increased thalamic activity leading to elevated thalamic input to the neocortex.^139^ Our data indicate that ACh may regulate this process, thereby increasing the gain of POm output. This could serve to amplify relevant sensory input during heightened attention and arousal.

L6b CT PCs exclusively provide direct facilitating synaptic input to the POm without recruiting the TRN neurons and thus do not engage this intrathalamic feedforward inhibitory circuit.^53^^,^^57^^,^^140^ This L6b-POm input is powerfully excited by orexin, a neuropeptide crucial for wakefulness and attention, which drives wake-like cortical states.^60^^,^^98^^,^^141^

The behavioral impact of L6b CT PCs has yet to be elucidated. Optogenetic activation of L6 DRD1+ neurons (putative L6b CT PCs) in S1 barrel cortex (BC) resulted in a transient increase in POm activity that has been hypothesized to play a role in motor cortex inhibition.^58^ In another study, BC L6b neurons were found to have distinct subcircuits targeting specific cortical layers and neurons, including L5 pyramidal neurons and different L6 interneuron types. Photoactivation of these neurons caused a robust enhancement of attention-associated high-gamma oscillations and neuronal population spiking while abolishing slow waves in sleep-deprived mice.^60^ This suggests that L6b neurons, despite being a small population, play a major role in the shift from wakefulness to attention, a finding that is in line with the high orexin density but also their strong responsiveness to ACh and dopamine, which also promote arousal and attention.

It has been proposed that CTC signaling acts not only as an intracortical feedback mechanism but also as a feedforward activation of adjacent cortical areas. For example, the recruitment of M1 cortex by S1 cortex via POm suggests that information transfer between cortical areas may occur indirectly.^142^ This indirect pathway is engaged by both deep L6a PCs and L6b PCs and is therefore subject to direct neuromodulatory control. It may be most effective during periods of arousal and attention.

In the entorhinal cortex (EC), L6b neurons primarily form synaptic contacts with hippocampal CA1 PCs. Optogenetic inhibition of this neuronal pathway affected spatial coding in CA1 PCs, whereas ablation of L6b neurons impaired both the acquisition of new spatial memories and the degradation of previously acquired ones. This indicates that EC L6b neurons are integral to sensory processing and memory storage at least in this cortical area.^143^

This study identifies three distinct FOXP2+ CT PC types in layer 6 that are tightly regulated by neuromodulators. Our findings reveal distinctive properties of these neurons, particularly L6b CT PCs, which are highly responsive to neuromodulators. The results underline the important role of L6b CT PCs in regulating the CTC feedback loop. By identifying specific subtypes of FOXP2+ CT PCs and their unique synaptic and neuromodulatory properties, this study advances our understanding of the neural mechanisms involved in sensory processing, attention, and arousal. It may also inform future research into therapeutic targets for neurological disorders involving corticothalamic dysfunction.

The distinct roles of L6a and L6b CT PCs in modulating thalamic activity through direct and indirect pathways highlight their importance in sensory processing under different behavioral states. L6a CT PCs modulate both excitatory and inhibitory thalamic circuits, potentially affecting sensory gating and attentional mechanisms. In contrast, L6b CT PCs provide direct excitatory input to the POm, which is modulated by ACh and dopamine. This input is critical for enhancing sensory input during wakefulness and attention. The division of labor within L6 CT PCs underscores the complexity of CT interactions and their relevance to adaptive sensory processing.

### Caveats and limitations of the study

The findings of this study provide evidence that FOXP2+ neurons in layer 6 of rodent somatosensory cortex are CT neurons and are highly susceptible to modulation by wakefulness-promoting neurotransmitters, such as ACh and dopamine, in particular those in layer 6b. A potential limitation of this study is that we did not investigate CT neurons from non-sensory cortical areas. Although FOXP2 immunoreactivity is high in layer 6 of virtually all neocortical areas,^37^^,^^144^ it is conceivable that subpopulations of L6 FOXP2+ neurons exist that have differential or no CT projections and are under less robust or differential neuromodulatory control. It was not feasible within the scope of this study to examine the behavioral consequences of neuromodulation on corticothalamic feedback. One limitation of our study is that we could not identify potential sex- and age-specific differences in our results because both young male and female animals were used. However, this represents a crucial avenue for future research. Moreover, the possibility remains that additional classes of CT neurons exist in layer 6, although the currently available evidence does not support this hypothesis. Further studies may seek to identify corticothalamic neuron types with potentially different dendritic and axonal morphologies and genetic marker expression profiles, as well as their functional roles.

## Resource availability

### Lead contact

Requests for further information and requests for resources and reagents should be directed to and will be fulfilled by the lead contact, Dr. Dirk Feldmeyer (d.feldmeyer@fz-juelich.de).

### Materials availability

This study did not generate new unique reagents.

### Data and code availability

• The paper does not report original code.
• All original electrophysiological, immunocytochemical, and retrograde tracing data reported and any additional information required to reanalyze the data reported in this paper are available from the lead contact upon request.
• Neuronal reconstructions in this study are standard ASC files and have been shared via “NeuroMorpho.Org (Feldmeyer archive)” in the key resources table.

## Acknowledgments

We would like to thank Werner Hucko for excellent technical assistance. We thank Dr. Karlijn van Aerde for custom-written macros in Igor Pro software and Dr. Manuel Marx for assistance in neuronal 3D reconstructions. We thank Dr. Xuan He and Prof. Dr. David Elmenhorst for the support in fluorescence imaging at the initial stage of this work.

We are grateful for funding support from the European Union’s Horizon 2020 Framework Programme for Research and Innovation under the Framework Partnership Agreement No. 650003 (HBP FPA) to D.F.

## Author contributions

G.Q., investigation, formal analysis, data curation, visualization, validation, methodology, and writing—review & editing.

D.Y., investigation, formal analysis, data curation, visualization, validation, methodology, and writing—review & editing.

F.M., investigation, formal analysis, visualization, validation, methodology, and writing—review & editing. A.B., investigation, formal analysis, validation, methodology, and writing—review & editing. F.Y., investigation, formal analysis, validation, methodology, and writing—review & editing. M.O., conceptualization, writing—review & editing, validation, supervision, funding acquisition, and resources. D.F., conceptualization, writing—original draft, writing—review & editing, validation, supervision, project administration, methodology, funding acquisition, and resources.

## Declaration of interests

The authors declare no competing interests.

## STAR★Methods

### Key resources table

**Table:** 

REAGENT or RESOURCE	SOURCE	IDENTIFIER
**Chemicals, peptides, and antibodies**		

Acetylcholine	Sigma-Aldrich	A6625
Dopamine	Sigma-Aldrich	D2960000
Atropine	Sigma-Aldrich	A0132
Mecamylamine	Tocris Bioscience	2843
Dihydro-β-erythroidine (DHβE)	Tocris Bioscience	2349
SKF81297	Tocris Bioscience	1447
Tetrodotoxin (TTX)	Sigma-Aldrich Roth	6973.1
Biocytin	Sigma-Aldrich Merck	B4261
Avidin-biotinylated horseradish peroxidase (Vector ABC staining kit)	Vector Labs	PK 6100
3,3-diaminobenzidine (DAB)	Acros Organics	32800 5000
Eukitt	Sigma-Aldrich	3989
Biocytin-conjugatged Alexa Fluor 594	Invitrogen	A12922
mouse-*anti*-NeuN primary antibody	Merck-Millipore	Cat# MAB 377; RRID: AB_2298772
Goat-*anti*-FOXP2 primary antibody	Santa-Cruz-Biotechnology	Cat# sc-21069; RRID: AB_21069
Chicken-*anti*-goat secondary antibody, Alexa Fluor 488	Thermo Fisher Scientific	Cat# A-21467; RRID: AB_2535870
Cholera toxin B (CTB-488) retrograde tracer	Molecular Probes	AF-488-CTB
Cholera toxin B (CTB-647) retrograde tracer	Molecular Probes	AF-647-CTB

**Experimental models: Organisms/strains**		

wild-type Wistar rats	Charles River	

**Software and algorithms**		

Patchmaster	HEKA Elektronik	
NEUROLUCIDA	Microbrightfield	
NEUROEXPLORER	Microbrightfield	
IGOR Pro 6	Wavemetrics	
MATLAB	MathWorks	
CellSens	Olympus	
ImageJ	Schneider et al.^145^; rsbweb.nih.gov/	
PlotsOfData	Postma and Goedhart^146^; https://huygens.science.uva.nl/PlotsOfData/	

### Experimental model and study participants details

Wild-type Wistar rats of both sexes (aged 16–24 postnatal days) were used in this study. Animals were purchased from Charles River, Germany and housed in the animal facilities of Forschungszentrum Jülich or the Max Planck Institute for the Neurobiology of Behavior. To determine single cell electrophysiological properties, 135 neurons from 42 animals were recorded. Synaptic physiology was determined in 48 neuronal connections from 29 animals and cholinergic and dopaminergic neuromodulation was investigated in 216 neurons from 64 animals. Morphological data were obtained from 64 neurons previously recorded in the electrophysiological experiments. Three animals were used for retrograde tracing and a further three were used for FOXP2 immunohistochemistry in whole slices of the neocortex. In total, 141 rats were used in this study.

All experimental procedures involving animals were performed in accordance with the guidelines of the Federation of European Laboratory Animal Science Association (FELASA), the EU Directive 2010/63/EU, and the German animal welfare law. An official licence for ‘*in vivo*’ experiments was granted by the ‘Landesamt für Natur-und Verbraucherschutz’ of the federal state of North-Rhine-Westphalia.

### Method details

#### Slice preparation

On the day of the experiment animals were anesthetized with isoflurane at a concentration <0.1% and decapitated. The brain was quickly removed and placed in an ice-cold modified artificial cerebrospinal fluid (ACSF) containing a high Mg^2+^ and a low Ca^2+^ concentration (4 mM MgCl_2_ and 1 mM CaCl_2_) to reduce potentially excitotoxic synaptic transmission during slicing; other components were identical to those in the perfusion ACSF as described below. To maintain adequate oxygenation and a physiological pH level, the solution was constantly bubbled with carbogen gas (95% O_2_ and 5% CO_2_). Thalamocortical slices^147^^,^^148^ were cut at 350 μm thickness using Leica VT1000S vibrating blade microtome and then transferred to an incubation chamber containing preparation solution for a recovery period of 30–60 min at room temperature before being transferred to the recording chamber.

#### Solutions

During whole-cell patch-clamp recordings, slices were continuously superfused (perfusion speed ∼5 mL/min) with ACSF containing (in mM): 125 NaCl, 2.5 KCl, 1.25 NaH2PO4, 1 MgCl2, 2 CaCl2, 25 NaHCO3, 25 D-glucose, 3 mho-inositol, 2 sodium pyruvate and 0.4 ascorbic acid, bubbled with carbogen gas and maintained at 30°C–33°C. Patch pipettes were pulled from thick-wall borosilicate glass capillaries and filled with an internal solution containing (in mM): 135 K-gluconate, 4 KCl, 10 HEPES, 10 phosphocreatine, 4 Mg-ATP, and 0.3 GTP (pH 7.4 with KOH, 290–300 mOsm). The ‘searching’ pipette was filled with an internal solution in which K+ is replaced by Na+ (containing (in mM): 105 Na-gluconate, 30 NaCl, 10 HEPES, 10 phosphocreatine, 4 Mg-ATP and 0.3 GTP), in order to prevent the depolarisation of neurons during searching for presynaptic neurons. Biocytin at a concentration of 5 mg/mL was added to the internal solution in order to stain patched neurons after recordings. In addition, biocytin-conjugated Alexa Fluor 594 dye (1:500, Invitrogen) was added to the internal solution for post hoc identification of patched neurons during fluorescence imaging.

#### Electrophysiological recording and analysis

The slices and neurons were visualised using an upright microscope equipped with an infrared differential interference contrast (IR-DIC) optics. The barrels in layer 4 (L4) of the somatosensory cortex can be identified as dark stripes with light ‘hollows’ at low magnification (4x objective) and were visible in 6–8 consecutive slices.^148^ For single-cell recordings, neurons throughout the entire layer 6, i.e.,. from the border with deep layer 5b to the WM were randomly selected.^52^^,^^149^ L6 pyramidal cells (PCs) and L6 interneurons can be distinguished by their soma shape an the presence or absence of an ‘upright’ apical dendrite at high magnification (40x objective). They can also be differentiated by their intrinsic action potential (AP) firing patterns and - following histological staining - by their morphology.

Whole-cell patch clamp recordings were made using an EPC10 amplifier (HEKA, Lambrecht, Germany). Signals were sampled at 10 kHz, filtered at 2.9 kHz using Patchmaster software (HEKA), and later analyzed offline using Igor Pro software (Wavemetrics, USA). Recordings were performed using patch pipettes of 5–8 MΩ resistance. To find synaptic connections we used a ‘searching procedure’ described previously.^148^^,^^150^ Briefly, after patching a ‘postsynaptic neuron’, potential presynaptic neurons were patched with a ‘searching’ pipette filled with a Na-based internal solution. When an AP elicited in ‘loose cell-attached’ mode resulted in an excitatory postsynaptic potential (EPSP) in the postsynaptic neuron, this presynaptic neuron was re-patched with a new pipette filled with biocytin-containing internal solution. EPSPs were recorded from a postsynaptic neuron by evoking 2–3 APs or a train of 10 APs in the presynaptic neuron with brief (5 ms) depolarising pulses at 10 Hz. The interval between the simulation trains was 10–20 s. For each synaptic connection, 40 or more sweeps were collected during recordings.

Custom-written macros in Igor Pro 6 (WaveMetrics, Lake Oswego, USA) were used to analyze the recorded electrophysiological signals. To assess passive and active action potential firing properties, a series of 1 s current pulses starting with initial hyperpolarisation, followed by depolarisation, were elicited under current clamp configuration. Neurons with a series resistance exceeding 40 MΩ or that displayed a depolarised membrane potential (>−55 mV) after the cell membrane was ruptured were excluded from the data analysis. The resting membrane potential (V_rest_) was recorded immediately after establishing the whole-cell recording configuration. Passive membrane properties, including the input resistance (R_in_), membrane time constant τm, and voltage sag, were measured by analysing membrane potential (V_m_) traces induced by a series of hyper- and depolarising subthreshold current pulses. Additionally, for the 1^st^ AP elicited by a rheobase current step, the threshold, amplitude, half-width, latency, and AHP amplitude and latency were determined as single action potential properties.

Properties related to repetitive firing, such as spike frequency adaptation, inter-spike intervals (ISIs), AP amplitude, AP half-width accommodation, and AHP change, were measured for a current step that elicited approximately 10 APs. The analysis of most electrophysiological parameters has been described previously^151^ with some exceptions. AHP latency was defined as the time interval between the AP threshold and AHP trough and AHP change was calculated as the voltage difference between the last AHP and the 1st AHP. The adaption ratio was calculated by measuring the last ISI relative to the 3^rd^ ISI (excluding ISIs of initial bursts, doublets, or triplets) and the standard deviation of ISIs (S.D. of ISIs) was calculated as the S.D. of ISI_3_, ISI_4_, …, ISI_9_.^47^^,^^152^ Synaptic properties were evaluated as described previously.^145^^,^^148^ All unitary EPSP (uEPSP) recordings were aligned to their corresponding presynaptic AP peaks, and the mean uEPSP was generated by averaging the recordings. The amplitude of the EPSP was calculated by subtracting the mean baseline from the maximum voltage of the postsynaptic event. The paired-pulse ratio was defined as the amplitude of the 2^nd^ uEPSP divided by that of the 1st uEPSP, which was elicited by presynaptic action potentials at a stimulation frequency of 10 Hz.

#### Drug application and analysis

Acetylcholine (ACh) at either low (30 μM) or high (1 mM) concentration and dopamine (DA, 30 μM) were applied via the perfusion system. Atropine (ATRO, 200 nM), mecamylamine (MEC, 10 μM), dihydro-β-erythroidine (DHβE, 10 μM), SKF81297 (1 μM) and tetrodotoxin (TTX, 0.5 μM) were all bath applied; drugs were purchased from Sigma-Aldrich or Tocris. For single-cell recordings, a stable baseline with fluctuation in V_m_ <1 mV was recorded for 3 min before drug application via the perfusion system. ΔVm was calculated as the difference between the peak V_m_ excursion (positive or negative) following drug application and the baseline potential. For paired recordings, 10–20 sweeps were recorded as baseline before drug application. Sweeps were continuously recorded during drug application (40–60 sweeps) and washout (80–100 sweeps).

#### Immunohistochemical staining and imaging

To determine the FOXP2 immuoreactivity in L6 neurons recorded in acute brain slices, slices (350 μm) were fixed after electrophysiological recordings with 4% paraformaldehyde (PFA) in 100 mM phosphate buffered saline (PBS) for at least 24 h at 4°C and then permeabilised in 1% milk power solution containing 0.5% Triton X-100 and 100 mM PBS. Primary and secondary antibodies were diluted in the permeabilisation solution (0.5% Triton X-100 and 100 mM PBS) shortly before experiments. For FOXP2 staining of patched neurons, slices were incubated overnight with Goat-*anti*-FOXP2 primary antibody (1:500, Santa Cruz Biotechnology) at 4°C and then rinsed thoroughly with 100 mM PBS. Subsequently, slices were treated with Alexa Fluor secondary antibodies (1:500) for 2–3 h at room temperature in the dark. After being rinsed in 100 mM PBS, slices were embedded in Fluoromount. Fluorescence images were taken using either the Olympus CellSens platform or a Zeiss Scope A1 microscope. Prior to antibody labeling, the position of the patched neurons was marked by addition of the biocytin-conjugated Alexa Fluor 594 dye to the intracellular solution (Figures 1C and 1D). Slices were then incubated in 100 mM PBS overnight and processed for subsequent histological staining for morphological reconstruction and analysis as described below.

For FOXP2 antibody labeling of whole-slices, thinner slices (150 μm) were prepared and processed following the same procedure as described above. To determine the percentage of FOXP2–immunoreactive neurons in cortical L6 and the POm, slices were treated with both antibodies for FOXP2 and the neuronal marker protein NeuN. The primary antibody used for NeuN staining was mouse-*anti*-NeuN (1:250, Merck-Millipore). Fluorescence images (single images and image stacks) from different cortical and subcortical brain regions were acquired at constant exposure times and at different magnification levels. To ensure comparability between the co-localisation of our target antigens FOXP2 and NeuN, we maintained the same settings, slice, region, and intra-stack image distance. Single images and image stacks were assessed using the freely available ImageJ software (National Institute of Health; http://rsbweb.nih.gov). Image stacks were first visualised by a 3D surface plot and further processed by a standard z-projection. FOXP2+ and NeuN+ neurons were counted with the aid of the cell counter plugin tool for ImageJ.^153^ Counts were averaged across all slices and analyzed to determine the fraction of cells in which FOXP2 and NeuN were co-localised.

To recover the morphology of biocytin-filled neurons or neuron pairs, slices were rinsed several times in 100 mM PBS and then treated with 1% H_2_O_2_ in PBS for about 20 min in order to reduce any endogenous peroxidase activity. Slices were rinsed repeatedly with PBS and then incubated in 1% avidin-biotinylated horseradish peroxidase (Vector ABC staining kit, Vector Lab. Inc., Burlingame, USA) containing 0.1% Triton X-100 for 1 h at room temperature. The reaction was catalyzed using 0.5 mg/mL 3,3-diaminobenzidine (DAB; Sigma-Aldrich, St.Louis, Mo, USA) as a chromogen. Slices were then rinsed with 100 mM PBS, followed by slow dehydration with ethanol in increasing concentrations and finally in xylene for 2–4 h. After that, slices were embedded using Eukitt medium (Otto Kindler GmbH, Freiburg, Germany).

#### Morphological reconstruction and analysis

Computer-assisted morphological 3D reconstructions of L6 neurons were made using NEUROLUCIDA software (MicroBrightField, Williston, VT, USA) and Olympus BV61 microscopy fitted with a 100x objective. Neurons were selected for reconstruction based on the quality of biocytin labeling when background staining was minimal. The cell body, dendritic and axonal branches were reconstructed manually under constant visual inspection to detect thin and small collaterals. Barrel and layer borders, pial surface and the white matter (WM) were delineated during reconstructions at lower magnification 4x. The position of soma and layers were confirmed by superimposing the DIC images taken during the recording. The tissue shrinkage was corrected using correction factors of 1.1 in the x–y direction and 2.1 in the z direction.^146^ Because the cortical thickness of slices where neurons were stained vary from slice to slice, individual neuronal reconstructions were normalised to the average thickness along the pia-WM axis before the morphological analysis. Parameters characterising general morphological properties of reconstructed neurons, e.g., the perimeter and area of somata, the number and length of axonal and dendritic branches, were extracted using the NEUROEXPLORER software (MicroBrightField Inc., Willston, VT, USA). Other morphological parameters have been described before^55^ except for the absolute and relative depth of somata: soma depth was calculated as the pia-to-soma distance along the pia-WM axis and the relative depth was calculate as the ratio of the pia-to-soma distance to the pia-to-WM distance.

Furthermore, the 3D density maps of axonal and dendritic length were obtained using computerised 3D reconstructions, where the length of the axonal and dendritic tree per unit volume of 50 × 50 × 50 μm3 was calculated. The soma center of each neuron was given the co-ordinates of X, Y, Z = (0, 0, 0), and the relative coordinate of the beginning and endpoint of each segment in the tracing were obtained using the segment point analysis in NEUROEXPLORER. Further steps were carried out in MATLAB (MathWorks, Cambridge, UK) using a custom-written algorithm. The 3D axonal and dendritic density maps were calculated for each representative reconstructed neuron from a single group. These were then averaged to obtain the 3D density map for this group. Individual density maps were aligned with respect to the barrel center. The averaged density map for each group was smoothed using the 3D smooth function in MATLAB with a Gaussian kernel (s.d. = 50 μm). Isosurfaces at the 80-percentile were calculated for the smoothed density maps. Finally, axonal and dendritic density maps were visualised after projecting to 2D or 1D using two different colors, e.g., blue and red, respectively.

#### Retrograde tracing in combination with FOXP2 immunolabelling

Male Wistar rats were provided by Charles River Laboratories. All experiments were carried out after evaluation by the local German authorities, and in accordance with the animal welfare guidelines of the Max Planck Society. Neurons in cortex were retrogradely labeled as described previously (Rojas-Piloni et al., 2017). Briefly, young (P22–P25) male Wistar rats were injected with 1 mg/mL buprenorphine SR (0.05mL subcuteneous) approximately 30 min prior to surgery, and then anesthetized with isoflurane/O_2_ gas mixture (2% isoflurane v/v). Rats were then placed in a stereotaxic frame (Kopf Instruments 1900) and given an injection of 0.25% bupivacaine (0.10cc, s.q.) at the incision site. Then a 5 cm incision across the midline was made to expose the skull. Both bregma and lambda were marked with a surgical pen. Two small craniotomies were made using a dental drill (Osada EXL-M40) over the injection sites of the right cerebral hemisphere. Injection site coordinates were as follows (in mm): POm: 2.1 lateral from midline, 3.25 posterior to bregma and 5.2 deep from the pia; VPM: 3.25 lateral from midline, 2.9 posterior to bregma and 5.5 deep from the pia. Prior to injecting tracers into the VPM and POm, the head of the rat was leveled with a precision of 1 μm in both the medial–lateral and anterior–posterior planes using an electronic leveling device (eLeVeLeR; Sigmann Elektronics, Hüffenhardt, Germany) mounted to an adapter for the Kopf stereotax. Retrograde tracers, CTB-488 and CTB-647 (Molecular Probes; 1 mg/mL in PBS) were pressure injected (50–200 nL) under visual control into VPM and POm thalamus, respectively, using a 30cc syringe coupled to a calibrated glass injection capillary. After injection of tracers, the incision site was thoroughly cleaned with saline and sutured. Rats underwent a 5–7-day incubation period after tracer injection before transcardial perfusion with 4% PFA dissolved in 100 mM PBS. Brains were then cut into consecutive 40 μm thick coronal sections and immunolabelled with NeuN or FOXP2 as described above. The injection sites in VPM and POm were validated on low-resolution images of the thalamus (Figure 2A). Quantification of retrogradely labeled neurons in cortex was performed on high-resolution confocal images (Figures 2B–2D). The images were acquired using a confocal laser scanning system (Leica Application Suite Advanced Fluorescence SP5; Leica Microsystems) with glycerol immersion objectives (HC PL APO 10 × 0.4 N.A., HC PL APO 20 × 0.7 N.A.), a tandem scanning system (Resonance Scanner: 8 kHz scanning speed), spectral detectors with hybrid technology (GaAsP photocathode; 8x line average) and mosaic scanning software (Matrix Screener, beta version provided by Frank Sieckmann, Leica Microsystems). The laser excitation/emission settings were: Alexa Fluor 488 (excitation: 488nm (Argon-laser); emission detection range (495–550 nm), Alexa Fluor 594 (excitation: 561 nm (DPSS-laser); emission detection range (600–630 nm), Alexa Fluor 647 (excitation 633 nm (HeNe-laser); emission detection range (650–785 nm). For CTB-488 and CTB-647 double injection experiments, done in combination with FOXP2 staining conjugated with Alexa 594, the images were taken sequentially using either the 10x objective with a digital zoom of 1.7 (0.868 × 0.868 μm per pixel), or a 20x objective with a digital zoom of 2.0 (0.361 × 0.361 μm per pixel).

### Quantification and statistical analysis

For all data, the mean ± standard deviation is provided. In order to assess significant differences between two independent samples that may not be normally distributed, a non-parametric Wilcoxon Mann-Whitney U test was performed. In order to compare paired data that were not normally distributed, the non-parametric Wilcoxon signed-rank test was employed. The level of statistical significance was set at *p* < 0.05, with n indicating the number of neurons/pairs analyzed. Boxplots were utilised whenever the sample size was ≥10. For this, the web application PlotsOfData^154^ (https://huygens.science.uva.nl/PlotsOfData/) was used.
