# Supplementary material for: FOXP2-immunoreactive corticothalamic neurons in neocortical layers 6a and 6b are tightly regulated by neuromodulatory systems
Source: iScience. 2024 Dec 19;28(1):111646. doi: 10.1016/j.isci.2024.111646 (PMC11758397; doi:10.1016/j.isci.2024.111646)
Supplement: Document S1. Figures S1–S8 and Tables S1–S3 [file mmc1.pdf]

iScience, Volume *28*

Supplemental information

FOXP2-immunoreactive corticothalamic neurons in neocortical layers 6a and 6b are tightly regulated by neuromodulatory systems

Guanxiao Qi, Danqing Yang, Fernando Messore, Arco Bast, Felipe Yáñez, Marcel Oberlaender, and Dirk Feldmeyer

FOXP2-immunoreactive corticothalamic neurons in neocortical layers 6a and 6b are tightly regulated by neuromodulatory systems

Guanxiao Qi[1]*, Danqing Yang[1,2]*, Fernando Messore[3,4], Arco Bast[3,4], Felipe Yáñez[3,5], Marcel Oberlaender[3,6] and Dirk Feldmeyer[1,2,7]

[1] Institute of Neuroscience and Medicine 10, Research Centre Jülich, 52425 Jülich, Germany

[2] Dept. of Psychiatry, Psychotherapy and Psychosomatic, RWTH University Hospital, 52074 Aachen, Germany

[3] In Silico Brain Sciences Group, Max Planck Institute for Neurobiology of Behavior – caesar, 53175 Bonn, Germany

[4] International Max Planck Research School (IMPRS) for Brain and Behavior, 53175 Bonn, Germany

[5] International Max Planck Research School (IMPRS) for Intelligent Systems, 72076 Tübingen, Germany

[6] Department of Integrative Neurophysiology, Center for Neurogenomics and Cognitive Research, Vrije Universiteit Amsterdam; 1081 Amsterdam, the Netherlands

[7] Jülich-Aachen-Research Alliance ʹBrain ʹ- Translational Brain Medicine

[*]these authors have contributed equally to this work

Correspondence:

Univ.-Prof. Dr. Dirk Feldmeyer

Forschungszentrum Jülich GmbH

Institut für Neurowissenschaften und Medizin 10 (INM-10)

Leo-Brandt-Strasse

52425 Jülich

Germany

e-mail: d.feldmeyer@fz-juelich.de

Lead Contact:

e-mail: d.feldmeyer@fz-juelich.de

Supplemental Figure Legend

Suppl. Figure S1. FOXP2 Immunoreactivity in the POm. 98.8±2.5% all POm neurons showed FOXP2 immunoreactivity. The left panel shows NeuN labelling, the middle panel the FOXP2 labelling, and the right panel an overlay of the biocytin and FOXP2 staining. The scale bar in the right panel represents the other two panels. Data were of obtained from nine slices in nine different animals.

Suppl. Figure S2. Relative somatic depth of recorded L6a FOXP2+ (n=35), L6a FOXP2– (n=27), L6b FOXP2+ (n=63) and L6b FOXP2– (n=34) excitatory neurons. Layer 6 comprises approximately one third of the entire cortical depth (between 65% and 100% from the Pia (0%) to the layer6/WM border (100%)); it is highlighted as the semi-transparent yellow zone. Layer 6a is located in the upper 2/3 while L6b in the lower third (between 88% and 100%) of L6.

Suppl. Figure S3. Gallery of L6a FOXP2+ (n=10) and L6a FOXP2– (n=10) excitatory neuron morphologies. Insets in the reconstructions illustrate the FOXP2 immunoreactivity (see Methods for details). In all insets, the left panel shows the fluorescent biocytin-conjugated Alexa 594 labelling of the recorded neuron, the middle panel displays the FOXP2 labelling, and the right panel an overlay of the biocytin and FOXP2 staining. The scale bar in the right panel represents 20 µm and applies to the other panels.

Suppl. Figure S4. Gallery of L6b FOXP2+ (n=10) and L6b FOXP2– (n=10) excitatory neuron morphologies. Insets in the reconstructions illustrate the FOXP2 immunoreactivity (see Methods for details). In all insets, the left panel shows the fluorescent biocytin-conjugated Alexa 594 labelling of the recorded neuron, the middle panel displays the FOXP2 labelling, and the right panel an overlay of the biocytin and FOXP2 staining. The scale bar in the right panel represents 20 µm and applies to the other panels.>

Suppl. Figure S5. L6 interneurons show no FOXP2 immunolabelling. (A) Morphological reconstruction of a L6 FOXP− fast spiking interneuron. (B) Ten L6 interneurons including 3 fast spiking and 7 non-fast spiking interneurons were examined for FOXP2 labelling. The left panel shows iocytin-conjugated Alexa 594 labelling of the recorded interneuron, the middle panel the FOXP2 labelling, and the right panel an overlay of the biocytin and FOXP2 staining. The scale bar in the right panel represents 50 M and applies also to the other panels. None of them was FOXP2 immunoreactive (see insets in A).

Suppl. Figure S6. L6b FOXP2– inverted pyramidal and multipolar spiny neurons show distinct electrophysiological properties. (A) Representative morphological reconstructions (upper) of a L6b inverted pyramidal cell and (B) a L6b multipolar spiny neuron (right), both of which are not FOXP2+ (bottom photographs). (C, D) Electrophysiological recordings of AP firing patterns from the same neurons shown in (A, B). (E) Box plots for electrophysiological properties of L6b inverted (n=17) and multipolar (n=13) spiny neurons. The box indicates the interquartile range (IQR), the whiskers

show the range of values that are within 1.5*IQR and a horizontal line indicates the median. P value was calculated using the Wilcoxon Mann-Whitney U test.

Suppl. Figure S7. L5b pyramidal cells were not retrogradely labelled by CTB injections in the VPM (left, green) but only in the POm (middle, red). The two L5b pyramidal neurons innervating the POm do not show FOXP2 immunoreactivity (right, blue). Individual retrogradely labelled pyramidal cells are marked by white open arrow heads.

Suppl. Figure S8. Differential cholinergic modulation of synaptic connections established by L6b FOXP2+ and FOXP2– excitatory neurons. (A) Morphology and FOXP2 immunoreactivity (insets) for a L6b FOXP2+ (left) and FOXP2– (right) neuron pair. (B) EPSPs (upper) recorded under control condition in postsynaptic neurons induced by three APs (lower) in presynaptic neurons from the same neuron pairs shown in (A). (C) Time course of 1st EPSP amplitude change during the bath-application of ACh (30 µM) recorded from the same neuron pairs shown in (A,B).

Supplemental Table Legend

Table S1. Statistical tests for the morphological properties of L6a and L6b FOXP2+ and FOXP2− excitatory neurons. P value was calculated using the non-parametric Wilcoxon-Mann-Whitney two-sample rank test between individual groups.

Table S2. Statistical tests for the electrophysiological properties of L6a and L6b FOXP2+ and FOXP2− excitatory neurons. P value was calculated using the non-parametric Wilcoxon-Mann-Whitney two-sample rank test between individual groups.

Table S3. Statistical comparison of the electrophysiological properties of inverted and multipolar L6b FOXP2– excitatory neuron subtypes. p value was calculated using the non-parametric Wilcoxon-Mann-Whitney two-sample rank test.

Suppl. Figure S1

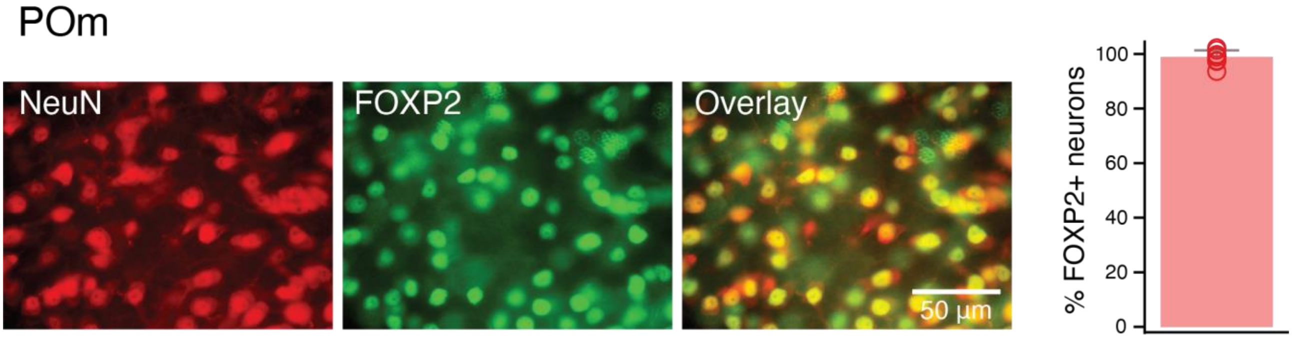

Suppl. Figure S2

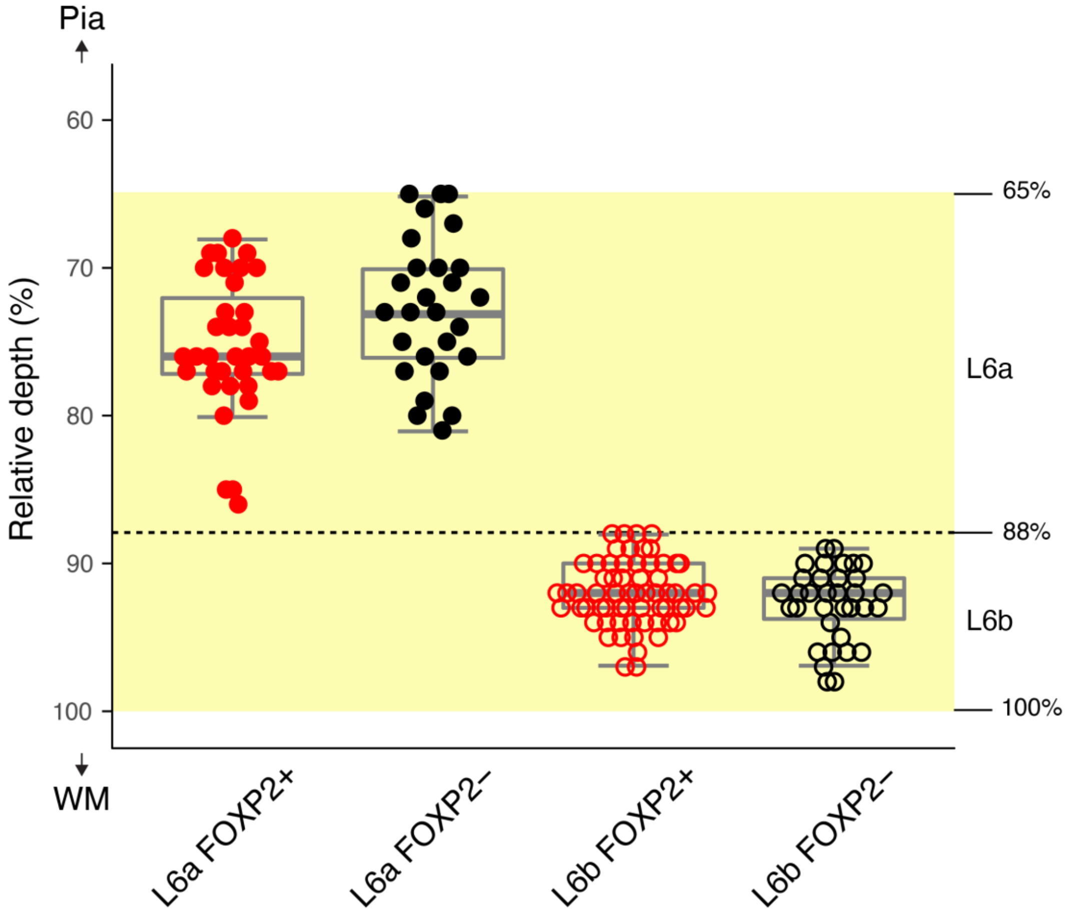

Suppl. Figure S3

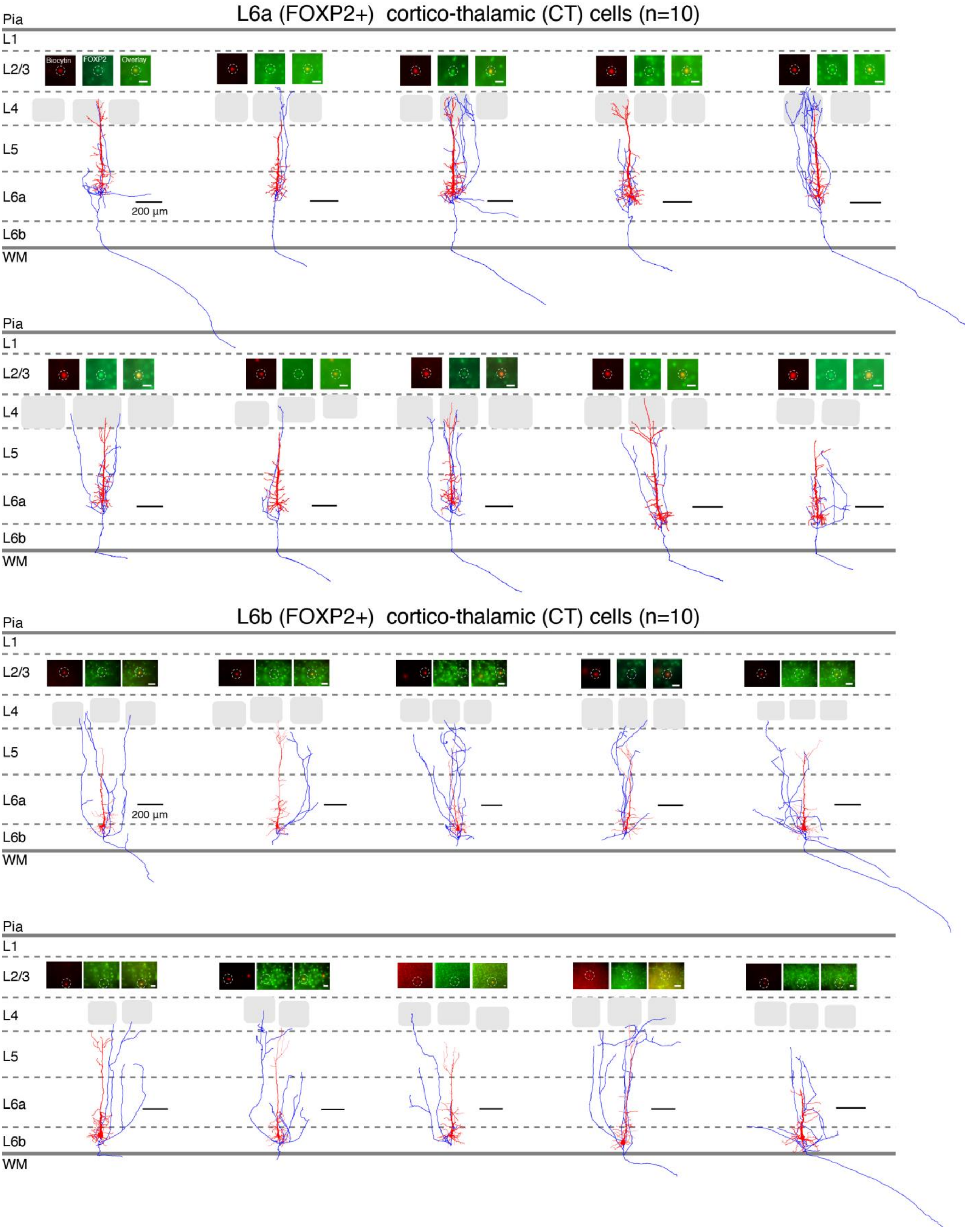

Suppl. Figure S4

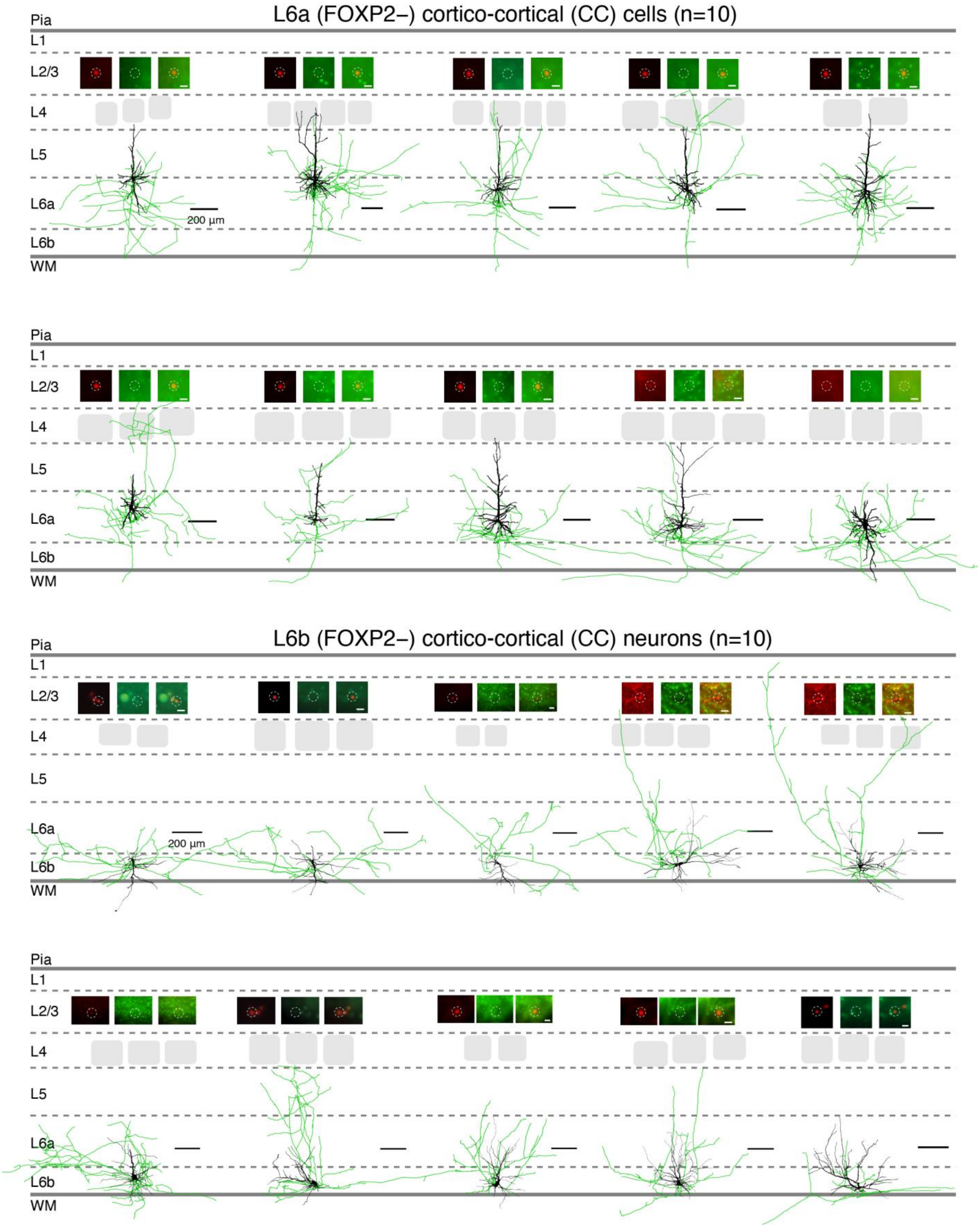

Suppl. Figure S5

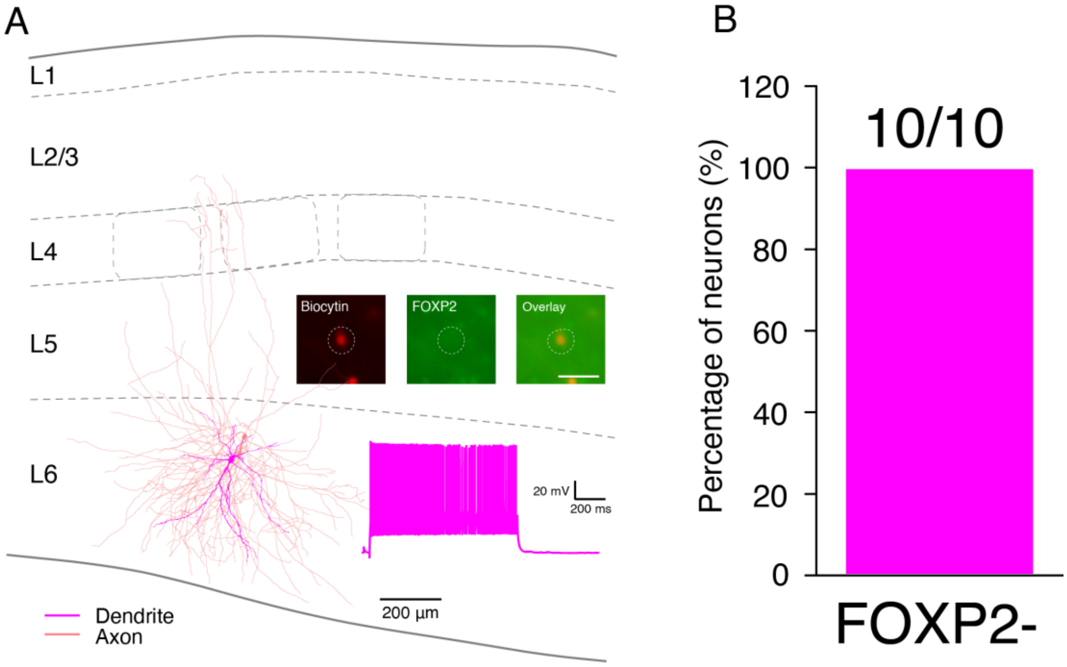

Suppl. Figure S6

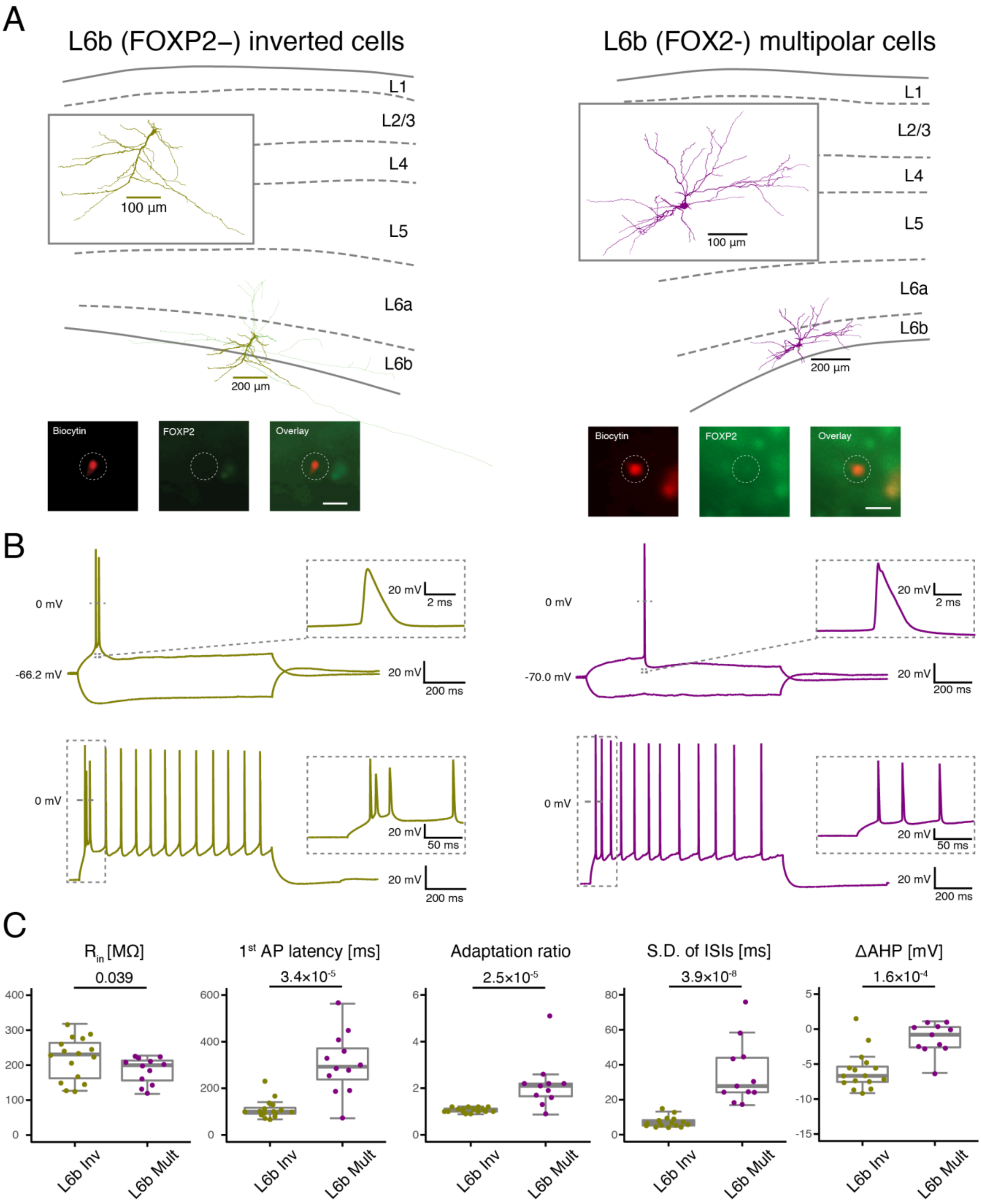

Suppl. Figure S7

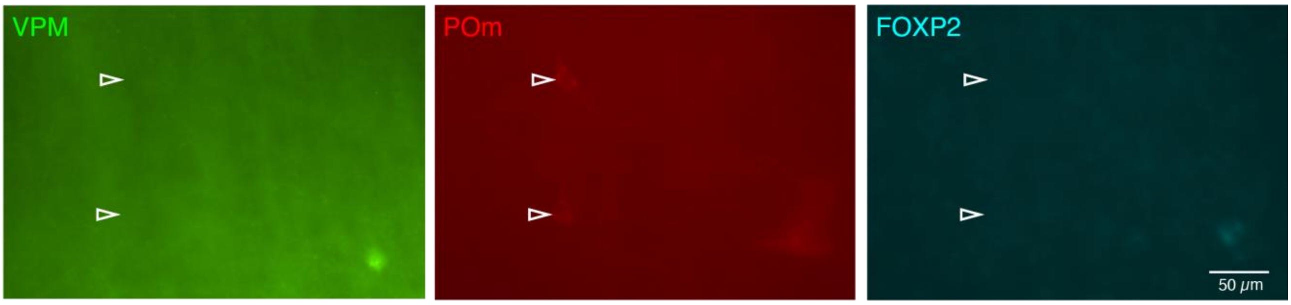

Suppl. Figure S8

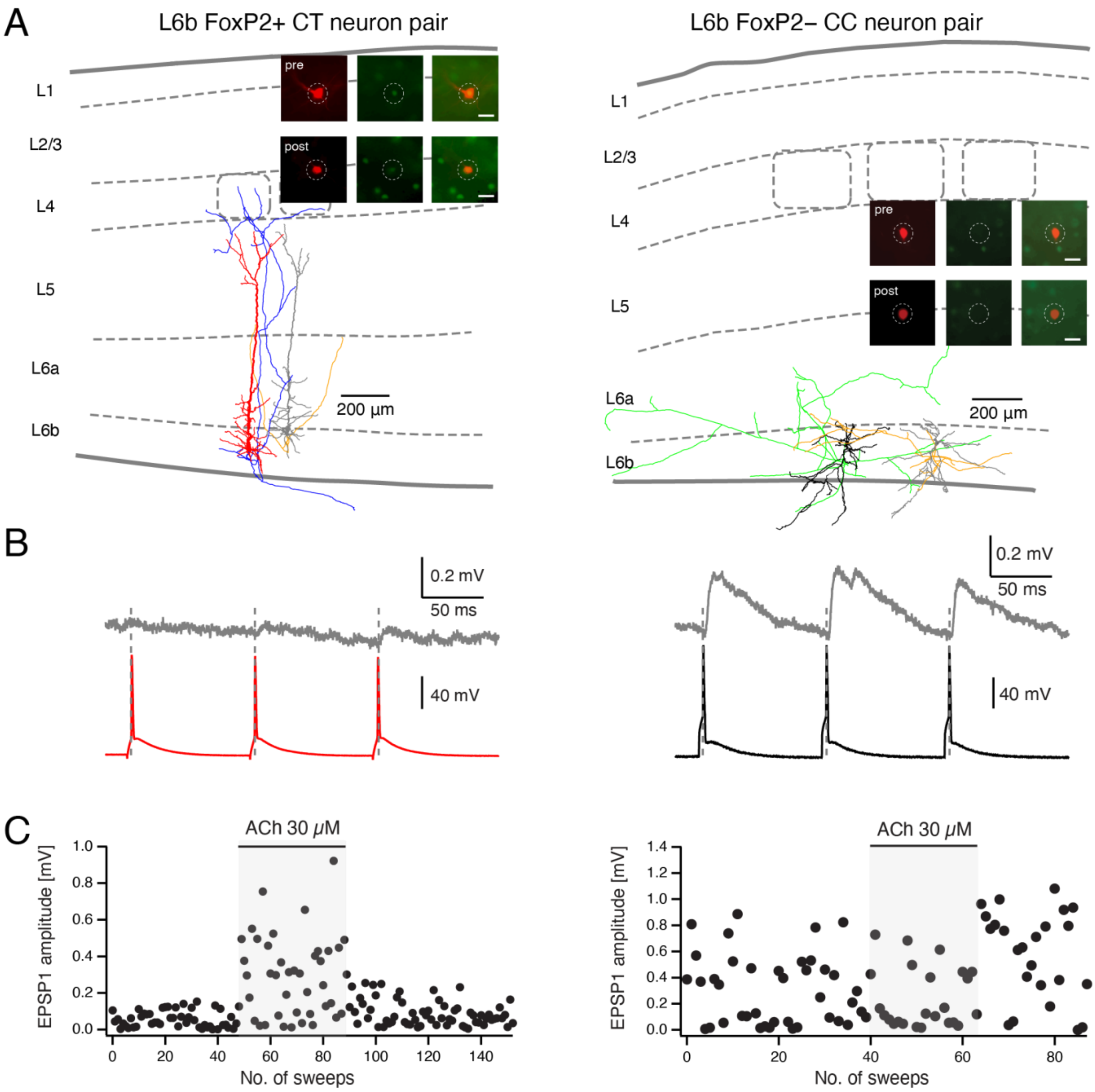

Supplemental Tables

Table S1

	Statistical significance			
	L6a FOXP2+ vs L6a FOXP2−	L6b FOXP2+ vs L6b FOXP2−	L6a FOXP2+ vs L6b FOXP2+	L6a FOXP2− vs L6b FOXP2−
Soma				
Depth (μm)	0.089	0.74	**2.88×10^{-3}**	**2.06×10^{-4}**
Relative depth (%)	0.083	0.14	**1.08×10^{-5}**	**1.08×10^{-5}**
Dendrite				
No. of apical dendrite branches	**1.94×10^{-3}**	N/A	0.30	N/A
Total length of apical dendrite (μm)	**8.93×10^{-3}**	N/A	0.39	N/A
No. of basal dendrites	0.36	0.098	0.42	1.06
No. of basal dendrite branches	0.18	**6.30×10^{-3}**	0.93	**0.03**
Total length of basal dendrites (μm)	**2.88×10^{-3}**	**2.17×10^{-5}**	0.58	**2.88×10^{-3}**
Horizontal field span of dendrite (μm)	**0.019**	**1.08×10^{-5}**	0.35	**2.06×10^{-4}**
No. of dendritic branches	**0.045**	0.14	0.42	0.34
Total length of dendrites (μm)	0.68	0.58	0.31	0.91
Axon				
Horizontal field span of axon (μm)	**1.08×10^{-5}**	**4.33×10^{-5}**	**0.019**	0.58
No. of axonal branches	**7.80×10^{-4}**	**1.40×10^{-3}**	0.45	**0.037**
Total length of axon (μm)	**4.33×10^{-5}**	**3.25×10^{-4}**	0.44	**0.011**

Table S1. Statistical tests for the morphological properties of L6a and L6b FOXP2+ and FOXP2− excitatory neurons. P value was calculated using the non-parametric Wilcoxon-Mann-Whitney two-sample rank test between individual groups.

	Statistical significance			
	L6a FOXP2+ vs L6a FOXP2−	L6b FOXP2+ vs L6b FOXP2−	L6a FOXP2+ vs L6b FOXP2+	L6a FOXP2− vs L6b FOXP2−
Passive				
Vrest (mV)	0.91	0.39	**0.02**	**1.59×10^{-3}**
Rin (MΩ)	0.058	0.75	**5.54×10^{-3}**	**9.51×10^{-4}**
Tau (ms)	**0.021**	0.14	**1.47×10^{-3}**	0.16
Sag (%)	0.24	**4.91×10^{-5}**	0.34	**9.72×10^{-3}**
Single AP				
Rheobase current (pA)	0.89	0.32	**2.71×10^{-3}**	**1.22×10^{-3}**
AP threshold (mV)	0.16	0.98	0.11	0.91
AP half-width (ms)	**2.19×10^{-8}**	0.32	**8.11×10^{-11}**	**9.72×10^{-3}**
AP amplitude (mV)	0.96	0.58	**0.017**	0.40
AP latency (ms)	**1.05×10^{-6}**	**2.23×10^{-3}**	**6.21×10^{-4}**	0.24
fAHP amplitude (mV)	**1.47×10^{-4}**	0.08	0.07	0.077
fAHP latency (ms)	**6.60×10^{-5}**	0.94	**4.51×10^{-11}**	**7.59×10^{-3}**
Repetitive firing				
Max. firing frequency (Hz)	0.24	0.69	**0.042**	0.32
Slope of F-I curve (APs/100	0.70	0.88	**0.046**	0.16
Adaptation ratio	0.31	0.22	0.66	0.70
s.d. of ISIs (ms)	**7.45×10^{-5}**	0.32	**2.64×10^{-4}**	0.057
ISI1 (ms)	0.12	**0.025**	**2.48×10^{-4}**	**2.67×10^{-6}**
ISI2 (ms)	**5.0×10^{-3}**	**0.024**	0.23	0.092
ISI3 (ms)	0.78	**0.034**	0.52	0.13
AP1 amplitude (mV)	0.52	0.37	0.10	0.46
AP2 amplitude (mV)	**4.87×10^{-7}**	0.11	**1.6×10^{-3}**	**6.89×10^{-4}**
AP3 amplitude (mV)	**0.028**	0.43	**0.021**	**0.013**
AP1 half-width (ms)	**1.06×10^{-8}**	0.35	**1.13×10^{-10}**	**0.02**
AP2 half-width (ms)	**7.64×10^{-7}**	**2.16×10^{-3}**	**1.14×10^{-10}**	0.36
AP3 half-width (ms)	**2.09×10^{-10}**	**8.07×10^{-3}**	**1.13×10^{-10}**	0.60
AP1 threshold (mV)	0.099	0.51	0.39	0.97
fAHP1 amplitude (mV)	**5.79×10^{-8}**	0.15	0.31	**2.04×10^{-3}**
AP9 threshold (mV)	0.32	0.75	0.31	0.84
fAHP9 amplitude (mV)	0.36	0.33	**7.60×10^{-3}**	**9.09×10^{-3}**
fAHP9 - fAHP1 (mV)	**6.53×10^{-11}**	0.055	**0.035**	**7.10×10^{-5}**

Table S2. Statistical tests for the electrophysiological properties of L6a and L6b FOXP2+ and FOXP2− excitatory neurons. P value was calculated using the non-parametric Wilcoxon-Mann-Whitney two-sample rank test between individual groups.

Table S2

Table S3

	L6b FOXP2– inverted (n=17)	L6b FOXP2– multipolar (n=13)	p value
Passive			
V_{rest} (mV)	-68.6 ± 6.8	-71.5 ± 6.0	0.28
R_{in} (MΩ)	221.5 ± 59.5	187.9 ± 37.1	**0.039**
τ_M (ms)	26.5 ± 5.6	21.2 ± 6.3	**0.017**
Sag (%)	7.9 ± 2.7	4.5 ± 3.9	**0.0091**
Single AP			
Rheobase current (pA)	96.5 ± 41.4	99.2 ± 33.2	0.61
AP threshold (mV)	-36.0 ± 4.1	-40.7 ± 3.9	**0.008**
AP half-width (ms)	1.02 ± 0.16	1.15 ± 0.22	**0.043**
AP amplitude (mV)	89.0 ± 6.8	97.7 ± 8.5	**0.0045**
AP latency (ms)	117.6 ± 49.1	302.8 ± 125.8	$\mathbf{3.41\times10^{-5}}$
fAHP amplitude (mV)	5.5 ± 3.5	11.9 ± 2.9	$\mathbf{2.00\times10^{-5}}$
fAHP latency (ms)	4.3 ± 0.9	24.5 ± 15.3	$\mathbf{5.00\times10^{-7}}$
Repetitive firing			
Max. firing frequency (Hz)	26.0 ± 8.2	19.5 ± 5.4	**0.012**
Slope of F-I curve (APs/100 pA)	18.1 ± 4.5	9.2 ± 4.4	$\mathbf{7.54\times10^{-5}}$
Adaptation ratio	1.1 ± 0.1	2.1 ± 1.0	$\mathbf{2.50\times10^{-5}}$
s.d. of ISIs (ms)	7.3 ± 3.0	36.4 ± 17.9	$\mathbf{3.85\times10^{-8}}$
ISI1 (ms)	11.7 ± 9.7	32.6 ± 10.7	$\mathbf{3.41\times10^{-5}}$
ISI2 (ms)	56.2 ± 33.0	43.9 ± 11.5	0.30
ISI3 (ms)	101.2 ± 22.1	52.1 ± 11.3	$\mathbf{1.66\times10^{-8}}$
AP1 amplitude (mV)	88.3 ± 6.4	96.1 ± 7.5	**0.0039**
AP2 amplitude (mV)	64.0 ± 9.5	91.2 ± 8.8	$\mathbf{1.17\times10^{-7}}$
AP3 amplitude (mV)	77.4 ± 7.8	90.2 ± 8.7	$\mathbf{4.95\times10^{-4}}$
AP1 half-width (ms)	0.96 ± 0.14	1.06 ± 0.23	0.094
AP2 half-width (ms)	1.64 ± 0.36	1.41 ± 0.38	0.10
AP3 half-width (ms)	1.47 ± 0.32	1.44 ± 0.40	0.80
AP1 threshold (mV)	-36.9 ± 4.0	-41.0 ± 5.0	**0.016**
fAHP1 amplitude (mV)	2.3 ± 3.9	6.3 ± 2.5	$\mathbf{7.34\times10^{-4}}$
AP9 threshold (mV)	-32.5 ± 4.5	-38.1 ± 5.4	**0.0013**
fAHP9 amplitude (mV)	12.5 ± 2.1	10.3 ± 1.7	**0.0051**
fAHP9 - fAHP1 (mV)	-5.84 ± 2.72	-1.11 ± 2.30	$\mathbf{1.62\times10^{-4}}$

Table S3. Statistical comparison of the electrophysiological properties of inverted and multipolar L6b FOXP2– excitatory neuron subtypes. p value was calculated using the non-parametric Wilcoxon-Mann-Whitney two-sample rank test.
